# Bidirectional backcrosses between wild and cultivated lettuce identify loci involved in nonhost resistance to downy mildew

**DOI:** 10.1007/s00122-018-3112-8

**Published:** 2018-05-25

**Authors:** Anne K. J. Giesbers, Erik den Boer, David N. J. Braspenning, Thijs P. H. Bouten, Johan W. Specken, Martijn P. W. van Kaauwen, Richard G. F. Visser, Rients E. Niks, Marieke J. W. Jeuken

**Affiliations:** 1 Plant Breeding, Wageningen University & Research, PO Box 386, 6700 AJ Wageningen, The Netherlands; 2 Present Address: Michelmore Lab, The Genome Center, Department of Plant Sciences, University of California, Davis, CA 95616 USA; 3 Present Address: Rijk Zwaan, 2678 ZG De Lier, The Netherlands; 4 Present Address: Limgroup, Veld Oostenrijk 13, 5961 NV Horst, The Netherlands; 5 Present Address: PAGV, Wageningen University & Research, Edelhertweg 1, 8219 PH Lelystad, The Netherlands

## Abstract

**Key message:**

The nonhost resistance of wild lettuce to lettuce downy mildew seems explained by four components of a putative set of epistatic genes.

**Abstract:**

The commonplace observation that plants are immune to most potential pathogens is known as nonhost resistance (NHR). The genetic basis of NHR is poorly understood. Inheritance studies of NHR require crosses of nonhost species with a host, but these crosses are usually unsuccessful. The plant-pathosystem of lettuce and downy mildew, *Bremia lactucae*, provides a rare opportunity to study the inheritance of NHR, because the nonhost wild lettuce species *Lactuca saligna* is sufficiently cross-compatible with the cultivated host *Lactuca sativa*. Our previous studies on NHR in one *L. saligna* accession led to the hypothesis that multi-locus epistatic interactions might explain NHR. Here, we studied NHR at the species level in nine accessions. Besides the commonly used approach of studying a target trait from a wild donor species in a cultivar genetic background, we also explored the opposite, complementary approach of cultivar introgression in a wild species background. This bidirectional approach encompassed (1) nonhost into host introgression: identification of *L. saligna* derived chromosome regions that were overrepresented in highly resistant BC1 plants (F1 × *L. sativa*), (2) host into nonhost introgression: identification of *L. sativa* derived chromosome regions that were overrepresented in BC1 inbred lines (F1 × *L. saligna*) with relatively high infection levels. We demonstrated that NHR is based on resistance factors from *L. saligna* and the genetic dose for NHR differs between accessions. NHR seemed explained by combinations of epistatic genes on three or four chromosome segments, of which one chromosome segment was validated by the host into nonhost approach.

**Electronic supplementary material:**

The online version of this article (10.1007/s00122-018-3112-8) contains supplementary material, which is available to authorized users.

## Introduction

Plants are generally resistant to most pathogenic organisms that they encounter. This is due to the narrow specialization of most pathogens: by far most pathogens have only a limited host range. The ability of a plant species to remain unaffected by all genotypes of a nonadapted pathogen is known as nonhost resistance (NHR) (Heath 2000; Thordal-Christensen 2003). The phenomenon in which microbe species try to establish a compatible interaction with a plant species is an ongoing evolutionary process with transitions from compatibility to incompatibility and the reverse. Therefore, some authors distinguish intermediate cases between host and nonhost status (Niks 1987; Bettgenhaeuser et al. 2014; Dawson et al. 2015).

NHR results from a continuum of layered defenses, constitutive and induced (Heath 1997; da Cunha et al. 2006; Ham et al. 2007). Current models of plant immunity state that NHR and host resistance involve the same components of the non-self-detection system: combined actions of basal immunity mediated by recognition of pathogen-associated molecular patterns (PAMPs) and of nucleotide-binding leucine-rich repeat (NLR) triggered immunity by recognition of pathogen effectors (Chisholm et al. 2006; Jones and Dangl 2006; Niks and Marcel 2009; Schulze-Lefert and Panstruga 2011). An often stated hypothesis for NHR, but so far without empirical evidence is that NLR immunity is relatively more important in interactions where the nonhost species is closely related to the normal host, whereas basal immunity would be more important in interactions involving a more distantly related nonhost species (Schulze-Lefert and Panstruga 2011). The importance of basal immunity for NHR could be mechanistically explained by a failure of the microbe’s effectors to effectively suppress basal immunity in nonhosts. In nonhosts, the cellular targets for the effectors may have diverged to an extent that hampers manipulation by the effectors.

Several approaches have been used to unravel the genetics of NHR in different plant-pathosystems. Most commonly, knock-out screenings on nonhost plants have been used to pinpoint genes that are necessary for the retention of resistance. (Collins et al. 2003; Lipka et al. 2005; Stein et al. 2006; An et al. 2016; Zhao et al. 2016). In contrast,  knock-out screenings on host plants have been used to identify host genes that are necessary for plant-pathogen compatibility and the retention of susceptibility (*S* genes) (Pavan et al. 2010; van Schie and Takken 2014).

A more direct way of identifying the responsible genes for natural variation of NHR would be genetic mapping in segregating populations (Niks and Marcel 2009). However, most host and nonhost species cannot be intercrossed, as most nonhost species are too much diverged from the host species to be cross-fertile.

One of the few plant-pathosystems in which host and nonhost species can be crossed is lettuce downy mildew. Lettuce (*Lactuca*) is a self-fertilizing diploid species (2*n* = 18). The nonhost species *Lactuca saligna* is cross-compatible with the cultivated host species *L. sativa*, though severely reduced F1 fertility, reduced F2 fertility and hybrid breakdown are common and reflect their genetic distance (De Vries 1990; Jeuken et al. 2001). Lettuce downy mildew is caused by *Bremia lactucae*, an obligate biotrophic oomycete, and leads to high yield losses in lettuce cultivation.

An interesting review about NHR in the context of evolutionary ecology by Antonovics et al. (2012) postulates two scenarios for nonhost resistance: “one-sided evolved” and “nonevolved”. The nonhost resistance of *L. saligna* may be “one-sided evolved” if *B. lactucae* did not counter-evolve to genetic changes in *L. saligna* that led to decreased infection levels. Alternatively, nonhost resistance in *L. saligna* could be “nonevolved” if the inability to infect *L. saligna* is a property of *B. lactucae* and not an evolved trait of *L. saligna,* for instance if *B. lactucae* specialized on another host like *L. serriola*. Generally, genetic variation for resistance in a nonhost is not expected, though may be incidentally present. Polymorphism for resistance is mainly expected for hosts that coevolved with a pathogen (Antonovics et al. 2012).

Histological analysis suggested that germinated conidia on *L. saligna* are arrested before normal hyphae formation (Lebeda et al. 2008; Zhang et al. 2009b). In previous genetic studies on progenies from *L. saligna* CGN05271 crossed with *L. sativa* cv Olof, we analyzed an F2 and a set of backcross inbred lines (BILs) that each contain one nonhost (*L. saligna*) introgression segment in a host (*L. sativa*) background (Jeuken and Lindhout 2004). In total, fifteen race-nonspecific minor or moderate QTLs were effective at different plant stages (Jeuken and Lindhout 2002; Jeuken et al. 2008;  Zhang et al. 2009a). Only two QTLs were effective during the entire lettuce life cycle (Zhang et al. 2009a). Stacking eight combinations of two QTLs did not result in greatly elevated levels of resistance (Den Boer et al. 2014). In conclusion, our earlier approach based on detection and stacking of individual QTLs did not lead to a combination of genes giving full resistance. Instead, NHR of *L. saligna* might be based on interactive, possibly epistatic, loci that have no individual effect but in combination lead to high levels of resistance. Such epistatic alleles should all be present in resistant segregants (F2 or backcross plants), whereas segregants with intermediate or susceptible phenotypes would not carry such a combination of epistatic alleles.

Some *L. saligna* accessions contain race-specific major gene resistances known as *R* genes (Parra et al. 2016; Giesbers et al. 2017). *R* genes typically encode NLR proteins and are mostly identified in resistant host plants. The race-specific *R* genes in *L. saligna* seem not essential for NHR, as segregating interspecific populations (F2s and BC1s backcrossed to the host) showed that segregants can be resistant while lacking such a monogenic dominant nonhost allele (Jeuken and Lindhout 2002; Giesbers et al. 2017). These resistant segregants may be explained by a combination of epistatic NHR loci.

This paper aims to broaden our insight into the genetics of NHR of the species *L. saligna* using bidirectional backcross populations. We addressed the following main questions: Is NHR based on presence of resistance factors from *L. saligna* or on absence of susceptibility factors from *L. sativa*? Do multiple *L. saligna* accessions share the same NHR genes? How many loci are essential for complete resistance? Through answering these questions, we have come a step closer to identifying the genes that cause NHR in *L. saligna*.

## Materials and methods

### Plant materials

Over a period of several years, we made many crossings with variable success rates between nine *L. saligna* accessions (nonhost) as a mother and *L. sativa* cv. Olof (host) as a father (Fig. S1). The latter is susceptible to all *B. lactucae* races and does not harbor any known *R* genes against *B. lactucae*. Eight *L. saligna* accessions were received from the Centre for Genetic Resources, The Netherlands (http://www.cgn.wur.nl): CGN05721, CGN05304, CGN05318, CGN05947, CGN11341, CGN15705, CGN15726, and CGN19047. One *L. saligna* accession from the island Corse, 275-5, was kindly provided by Dr. A. Beharav from the University of Haifa, Israel. The geographic origin of all accessions is listed in Table S1. F1 plants were all derived from a cross between a single mother and a single father plant. F1 plants as a mother were crossed to *L. sativa* cv. Olof as a father to develop for each of the nine *L. saligna* accessions a BC1 progeny, indicated as BC1cult (followed by an underscore and accession number to indicate the *L. saligna* parent) (Fig. 3b). Only the F1 from CGN05271 was also backcrossed to *L. saligna* CGN05271 as the father, indicated as BC1wild. This BC1wild population was further inbred for three generations until BC1wildS3 (Fig. 3c). In the generations BC1wildS1 and BC1wildS2, individuals were selected for enhanced infection severity compared to the previous generation (Relative Infection Severity > 0% in BC1wildS1 and > 10% in BC1wildS2) (see crossing scheme Fig. 3c).

An F2 population of *L. saligna* CGN05271 × *L. sativa* cv Olof (*n* = 126) was genotyped and used for linkage analysis to calculate genetic distances.

The following control lines were included in most disease tests: *L. sativa* cv Iceberg and dBIL468 (high levels of quantitative resistance at all plant stages except seedling stage (Zhang et al. 2009b; Den Boer et al. 2014)), BIL8.2 (low level of quantitative resistance at all plant stages except seedling stage) and *L. sativa* cv Olof, cv Cobham Green or occasionally cv Norden (susceptible). BIL268 and *L. sativa* cv Grand Rapids (high levels of quantitative resistance at young plant stage (Zhang et al. 2009b) were included in the histological analysis and in the young plant disease test.

### Host–nonhost classification of *Lactuca* species

Data of seedling infection severity to multiple (*n* = 36) *B. lactucae* isolates of *L. sativa* (1154 accessions), *L. serriola* (639 accessions), *L. virosa* (58 accessions) and *L. saligna* (55 accessions) were gathered from the Centre for Genetic Resources (http://cgn.websites.wur.nl/Website/downloads/DownloadCnr06.htm, accessed September 29, 2017). *B. lactucae test* isolates included Bl:1–7, Bl:10–26, UPOV set S1, SF1, IL4, CS9 and TV, and seven races collected from *L. serriola* (Lebeda 1986). We additionally tested seedlings of *L. saligna* 275-5, CGN11341 and CGN15726 with Bl:21, Bl:24 and Bl:29, as the CGN dataset lacked those infection severity data. Seedling infection severity was originally scored on a discrete 1 (resistant) to 9 (susceptible) scale. In a few cases, data were not scored on a 1–9 scale, but only on a 0, 3, 6, 9 scale (0 = complete resistant. 3 = incomplete resistant 6 = incomplete susceptible. 9 = susceptible). Score 0 was changed into score 1 for comparison between experiments. Details of the screening are described in Van Treuren et al. (2011). The numerical scores of each *B. lactucae* isolate were averaged per accession by taking the average of experiments scored on a 1–9 scale (experiments scored on a 0, 3, 6, 9 scale were only included if no data on a 1–9 scale were available), after which the resulting values ranging from 1.0 to 9.0 were transformed to a scale ranging from 0 to 100%, using the formula: infection severity level = (score − 1)/8 × 100.

### *L. saligna* phenogram

A phenogram of 73 *L. saligna* accessions was based on 423 AFLP fragments, derived from eight primer combinations. Distances were calculated using the “dist” function in the R package “stats” (R Core Team 2016). A tree was obtained using the neighbor-joining method in R package “ape” (Paradis et al. 2004). Three *L. sativa* cultivars represented the outgroup.

### Disease tests with *B. lactucae*

#### Seedling disease test

Seven to 10 days after seedling emergence 8–16 seedlings per lineage/genotype were inoculated with 2–4 × 10^5^ spores/ml of *B. lactucae* Bl:21, and for some also Bl:24 and Bl:29. Infection severity level (ISL) was scored at 7–12 dpi as the percentage of cotyledon area covered with sporulation, per cotyledon.

#### Histology and young plant disease test (YDT)

Nine 19-day-old plants per genotype (*L. saligna* 275-5, CGN05271 and CGN11341) were inoculated with 1.1 × 10^5^ spores/ml of *B. lactucae* Bl:21, as described by Zhang et al. (2009a). Six leaf samples per genotype were stained 48 h post-inoculation as described by Zhang et al. (2009b) and Van Damme et al. (2005). Developmental stages of the pathogen were counted per leaf sample. Five different types of infection units (IUs) were discerned: IU with only a primary vesicle (PV), IU that formed a secondary vesicle (SV), IU that formed intercellular hyphae (IH), IU with malformed hyphae (MAL-HY) and IU with haustoria (HA). Pictures showing these different developmental stages of *B. lactucae* are shown by Zhang et al. (2009b). Macroscopic ISL was scored visually as the percentage of leaf area covered with sporangiophores 10 days after inoculation on the two youngest fully expanded leaves at the moment of inoculation.

#### Adult plant disease test (ADTg)

Plants with hybrid necrosis symptoms (HN), observed as necrotic spots on leaves and associated with resistance, were excluded from disease phenotyping. Mostly HN symptoms can be observed easily at a macroscopic level. Incidentally, plants with less clear HN symptoms were classified and excluded based on genotypic data [markers at two loci, LG8 and LG9 (Jeuken et al. 2009)]. Independent detached leaf assays were conducted on 5–7-week-old adult plants as described by Jeuken and Lindhout (2002). Four leaf squares, each from a different fully extended leaf, were taken per test plant. Leaf squares (2 by 2 cm) were inoculated with 2–4 × 10^5^ spores/ml of *B. lactucae*. Infection severity level (ISL) was scored visually as the percentage of leaf area covered with sporangiophores between 10 and 14 days after inoculation.

#### Relative infection severity

Relative infection severity (RIS) levels were calculated as percentage relative to the absolute infection severity level (ISL) of the susceptible parent *L. sativa* cv. Olof. Plants with RIS ≤ 10% were considered as highly resistant.

#### Statistical analysis

To improve data normality, the percentage data of the F1 adult plant disease test were arcsine-root-transformed. General Analysis of Variance (ANOVA) was performed in GenStat 18, with genotype*plant as treatment structure. Predicted mean RIS values per line were compared in a Bonferroni test (*p* = 0.05).

### DNA isolation, DNA markers and genotyping

DNA was isolated from plant leaf tissue either by a high-throughput NaOH method (Wang et al. 1993) or by a modified CTAB method (Jeuken et al. 2001). For genotyping we used EST-based markers and Kompetitive Allele Specific PCR (KASPar) markers based on SNPs between *L. sativa* and *L. saligna*. The SNPs were obtained by mapping Illumina paired-end reads from *L. sativa* cv. Olof and a pool of five *L. saligna* accessions (CGN05304, CGN05318, CGN15705, CGN15726 and 275-5) against *the L. sativa* cv. Salinas genome version 8 (Reyes-Chin-Wo et al. 2017) using BWA-mem, version 0.6.3 (Li and Durbin 2009), with default settings. SNP calling was performed using Freebayes, version v1.0.2-29 (Garrison and Marth 2012) with default parameters. Subsequently the SNPs were filtered with SNPsift version 4.3 (Cingolani et al. 2012), with parameters: RPL&RPR > 1, SAF&SAR > 1, PAIRED&PAIREDR > 0.8, 6 < DP > 20, isHom&isRef for the *L. sativa* cv. Olof reads and isHom&isVariant for the *L. saligna* pooled reads. Flanking sequences were checked against the reference genome (*L. sativa* v8) using BLASTn (Altschul et al. 1990) to select for unique sites. The criterion for a SNP was: a same base for cv Salinas and cv Olof and the same alternative base in all reads of *L. saligna* accessions. From a collection of 9000 identified SNPs, we selected 293 genome-wide SNPs (with an average distance of 3.7 cM between markers) and seven chloroplastic SNPs for KASPar assays.

We distinguish three genotyping procedures:

1. All individuals of BC1cult_CGN05271, BC1wild_CGN05271 and BC1cult_CGN15705 were genotyped with 79, 83 and 77 EST-based markers respectively, more or less evenly distributed across the genome (Table S2). Polymorphisms between PCR products of *L. saligna* and *L. sativa* alleles were visualized by high-resolution melting curve differences on a LightScanner System (Den Boer et al. 2014).
2. BC1cult populations with extremely right  skewed distributions for infection levels to *B. lactucae* were selectively genotyped. They contained a high proportion (> 20%) of highly resistant plants (RIS ≤ 10%). These distributions resembled the segregation of a dominant monogenic *R* gene (50% complete resistant under normal segregation) in combination with segregation for quantitative resistance. To test whether and how many of these resistant plants were explained by dominant monogenic *R* genes, we genotyped these resistant plants with EST markers until a high percentage of co-segregation between linked EST markers and the resistance phenotype was observed. Co-segregation of full resistance and a *L. saligna* allele at one locus, indicating an *R* gene, was observed and described previously for three BC1 populations (CGN05304, CGN05318, CGN05947) in Giesbers et al. (2017). Plants for which the very low infection level was explained by an *R* gene were excluded from further NHR genotyping experiments.
3. The remaining highly resistant BC1cult plants (*n* = 32) from six *L. saligna* accessions were genotyped with 300 SNP-based genome-wide KASPar markers by Dr. Van Haeringen Laboratorium B.V., Wageningen, the Netherlands. KASPar SNP positions with 150 surrounding base pairs are listed in Table S3. The 300 SNP-based genome-wide genotyping further included the following plants: cross parents and their F1s (as controls), BC1wildS3 plants and their ancestors (back to three generations), an F2 population (*n* = 126) (Jeuken et al. 2001), and a set of backcross inbred lines (Jeuken and Lindhout 2004) from reference cross *L. saligna* CGN05271 × L. sativa cv. Olof.

### Detection of NHR regions

The observed genotypic ratio (heterozygous: homozygous host) of highly resistant BC1cult plants was compared to the expected Mendelian ratio of 1:1 for each marker using Chi-square tests. At least 18 independent genomic regions are expected, assuming that each of the nine linkage groups contains at least two independent regions due to one crossover per chromosome arm. To correct for multiple-testing, a genome-wide significance threshold of 0.05/18 = 0.003 was applied to obtain a genome-wide error rate of *p* = 0.05. Resistant BC1cult plants were tested together and per geographic subset (Israel, Southwest Asia, Europe). To exclude the possibility that one accession dominated the outcome of all tested plants, we also tested subsets: all BC1cult plants minus all plants of one accession (with more than five individuals). Subsequently, the identified loci with an overrepresentation of *L. saligna* alleles were compared with distorted segregation loci in genotyped BC1cult and BC1wild populations without phenotypic selection. In the BC1wildS3 lineages with enhanced susceptibility, introgression segments that were fixed for the homozygous *L. sativa genotype* were considered as regions nullified for NHR.

### Genetic map

KASPar markers were added to our latest F2 genetic linkage map (based on EST and AFLP-markers) from the cross *L. saligna* CGN05271 × *L. sativa* cv Olof (Jeuken et al. 2001). Linkage analyses were performed using JoinMap v5 software (Van Ooijen 2006). A new consensus genetic linkage map was calculated per linkage group using regression mapping and Kosambi’s mapping function with default settings: linkages with a recombination frequency smaller than 0.40, LOD scores higher than 1, a jump threshold of 5 and a third round. Marker intervals for studied traits in all populations were based on this F2 consensus map. Physical map locations refer to the *L. sativa* cv. Salinas reference lettuce genome v8 (Reyes-Chin-Wo et al. 2017; https://lgr.genomecenter.ucdavis.edu/). Here, we use the linkage group numbering and orientation of that reference *L. sativa* physical map, which differs from the numbering used in our previous publications. In order to relate previously reported gene and marker locations to the mapped loci in the current study, we present a conversion table (Table S4).

### Data availability

The dataset of Illumina raw reads for *L. sativa* cv. Olof and for pooled *L. saligna* accessions is available through NCBI Short Read Archive (BioProject ID PRJNA434185).

## Results

### Host–nonhost classification of *Lactuca* species

The broad resistance spectrum of *L. saligna* was described in a couple of studies since 1976 (Netzer et al. 1976; Norwood et al. 1981; Bonnier et al. 1992), but has not been graphically visualized. To illustrate the host and nonhost classification in *Lactuca* species, we have visualized a large dataset obtained from the Dutch Centre for Genetic Resources (CGN) of four *Lactuca* species and their *B. lactucae* infection scores at seedling stage, supplemented with some seedling disease test data for three accessions not tested by CGN.

**Fig. 1:**
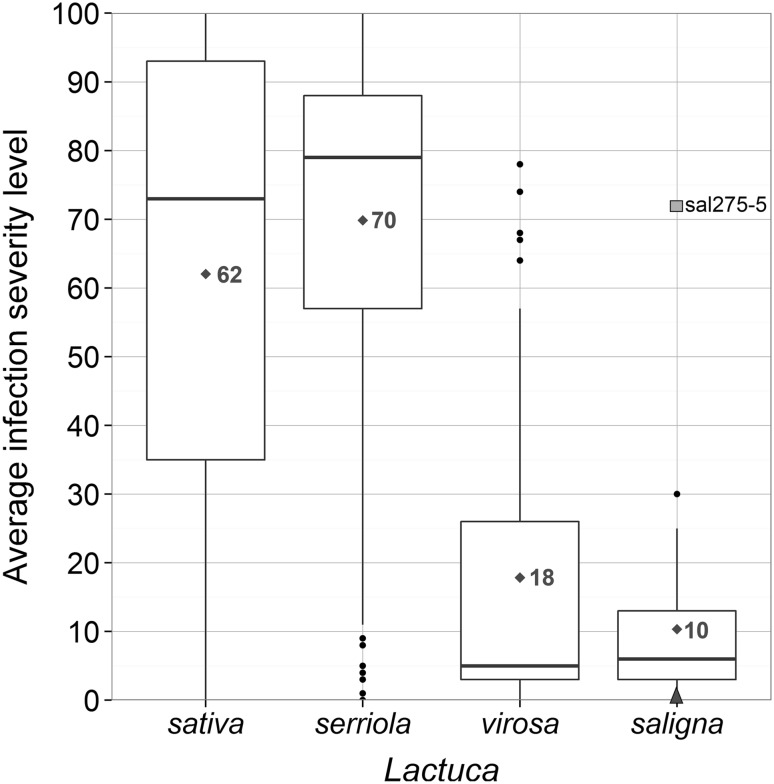
Boxplot of average infection severity level (%, at seedling stage) per *Lactuca* species based on public data of the Centre for Genetic Resources, the Netherlands (CGN) supplemented with our own data on three *L. saligna* accessions. The band inside the box is the median. Red numbers and diamonds indicate the species average. Averages are based on all tested interactions per accession tested with 10–23 Bl: races and for *L. saligna* also include the three additional accessions tested with only three races. Number of accessions included per species: *L. sativa* (*n* = 780)*, L. serriola* (*n* = 490)*, L. virosa* (*n* = 45), and *L. saligna* (*n* = 54). Green square (our data): *L. saligna* 275-5 average infection severity level to three Bl: races (21, 24 and 29). Blue triangle (our data): *L. saligna* CGN11341 and CGN15726 average infection severity level to three Bl: races (21, 24 and 29) (color figure online)

*L. sativa* (cultivated lettuce) and *L. serriola* (wild lettuce from the primary gene pool) have a high average infection severity level (ISL) of 62 and 70%, respectively, and are classified as a host species (Fig. 1). Most lines and accessions show either very low or very high ISL to individual *B. lactucae* races (heat map, Fig. S2ab). Resistances are explained mainly by the presence of one or more race-specific monogenic dominant *R* genes, and occasionally by some additional QTLs (Parra et al. 2016). *L. serriola* accessions show more intermediate interactions than *L. sativa* lines, possibly due to a higher frequency of minor genes for resistance.

*L. virosa* (wild lettuce from the secondary gene pool) has a low average ISL (18%, Fig. 1). Most accessions are highly resistant to all *B. lactucae* races, but several accessions show high levels of ISL (Fig. S2c). Therefore, at the species level *L. virosa* seems neither a true host nor a true nonhost. Not much is known about the genetics of resistance in *L. virosa*, except for the presence of a few *R* genes (Parra et al. 2016).

*L. saligna* (wild lettuce from the secondary gene pool) has on average a very low ISL (10%, Fig. 1). One exception is *L. saligna* 275-5 tested with three *B. lactucae* races (average ISL 72% at seedling stage, relative infection severity (RIS) 17% at adult plant stage). This *L. saligna* accession with unusually high infection at seedling stage was first reported by Petrželová et al. (2011), and we included it in our NHR study. Based on phenetic analysis (Fig. 3a), accession 275-5 fits in the European *L. saligna* clade. We consider *L. saligna* as a nonhost, despite the exception of this one accession.

### Multiple single-dose *L. saligna* alleles already result in low levels of infection severity

**Fig. 2:**
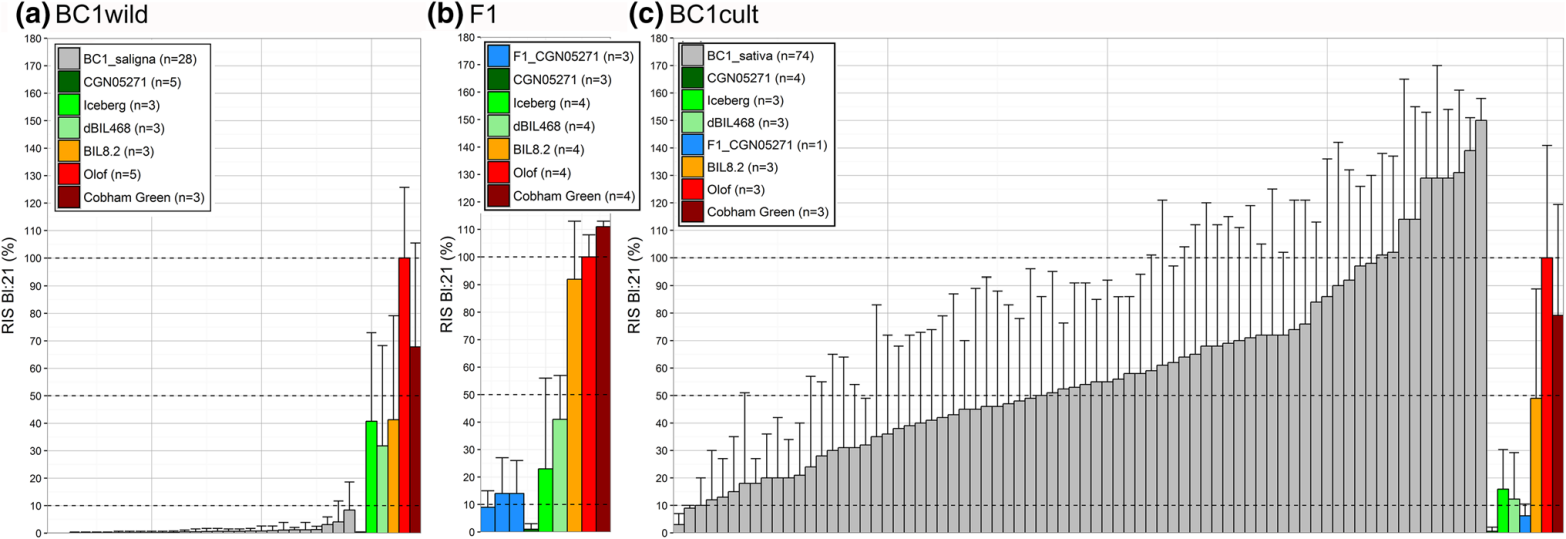
Relative infection severity levels to Bl:21 at adult plant stage in three independent experiments, including disease test controls (see insert boxes). **a** BC1wild (*n* = 28), **b** F1 (*n* = 3); **c** BC1cult (*n* = 74). For the test plants (BC1wild, F1 and BC1cult), averages are shown per individual plant. For the control lines, averages from multiple plants are shown

**Fig. 3:**
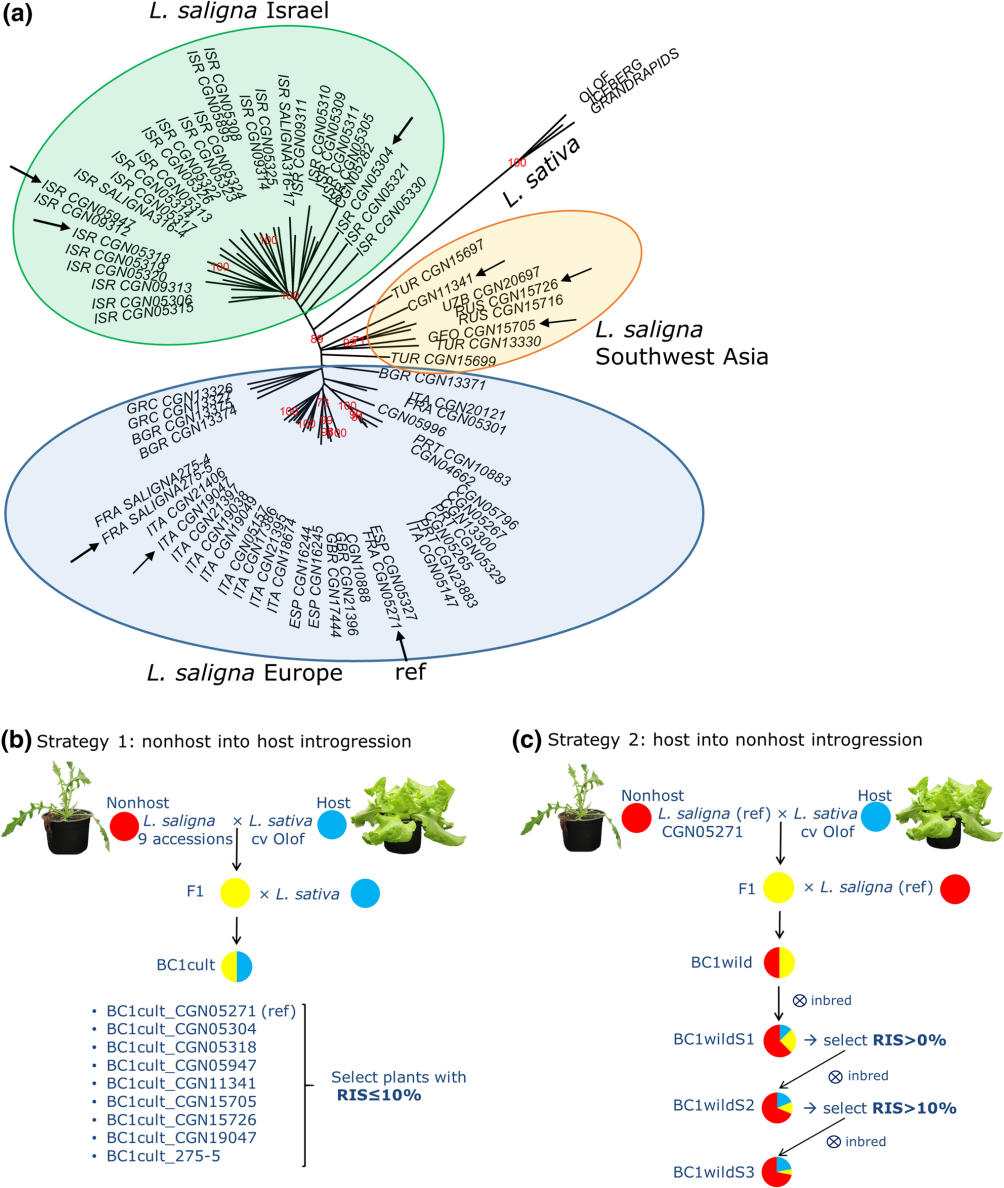
Selected *L. saligna* accessions and crossing scheme to obtain F1 plants and BC1 populations. Pie charts depict the average genotypic composition of each generation. Red: homozygous *L. saligna*, blue: homozygous *L. sativa*, yellow: heterozygous. **a** Unrooted neighbor-joining tree constructed of 423 AFLP fragments from 73 *L. saligna* and 3 *L. sativa* accessions. Three main branches are distinguished: European, Israeli and Southwest Asian. Bootstrap values greater than 60% (based on 1000 replicates) are indicated in red at the nodes. Arrows point to the nine selected accessions. **b** Each selected *L. saligna* accession was crossed with susceptible *L. sativa* cv. Olof (*L. sativa* ref), F1 plants were backcrossed to *L. sativa* cv. Olof resulting in BC1cult populations. **c** An F1 from *L. saligna* CGN05271 (*L. saligna* ref) was backcrossed to its *L. saligna* parent to obtain a BC1wild population and an inbred BC1wildS1 population. Subsequently, additional inbreeding was performed on plants which showed sporulation in disease tests (BC1wildS1: RIS > 0%, BC1wildS2: RIS > 10%) (color figure online)

To get an impression of NHR required gene and allele dosages, we compared infection levels of F1 generation and bidirectional backcross populations of the reference accession *L. saligna* CGN05271, crossed to the susceptible *L. sativa* cv Olof (Fig. 2). The F1, designated as F1_CGN05271, was backcrossed bidirectionally, to its resistant *L. saligna* parent and to its susceptible *L. sativa parent*, resulting in two distinct BC1 populations that we designated as BC1wild and a BC1cult, respectively (Fig. 3).

F1_CGN05271 showed a low infection severity level (average RIS 12%), similar to or lower than our quantitative resistant controls Iceberg and dBIL468 (Fig. 2b). All BC1wild plants (*n* = 28) showed a very low infection severity level (average RIS 1%, Fig. 2a). The low infection level of the F1 and BC1wild show that a single dosage of host (*L. sativa*) alleles on multiple loci does not lead to infection levels as high as the susceptible parent. The BC1cult population showed a continuous distribution from no infection to an infection severity similar to or higher than the *L. sativa* host*–*parent (mean RIS 59%, Fig. 2c). A small proportion (7%) of BC1cult plants showed infection severity levels similar to or lower than the F1. This BC1cult result indicates that a combination of nonhost (*L. saligna)* alleles, each in a single dose (heterozygous state), can already result in low infection severity.

These results indicate that NHR is based on resistance genes from *L. saligna* and not on absence of dominant susceptibility alleles from *L. sativa* (*S* genes). In the latter case, we would have expected all F1 and BC1cult plants to show a high infection severity and only a proportion of BC1wild plants with high infection severity levels. Instead, all BC1_wild plants showed very low infection severity levels. Infection levels in F1 and bidirectional BC1 populations suggest that identification of NHR loci can be done by selective genotyping of resistant plants (RIS ≤ 10%) in a BC1cult population, and by selective genotyping in inbred generations of BC1wild if increased infection phenotypes are observed.

### Strategies to dissect NHR of *L. saligna*

On the basis of these findings, we denominated two strategies of selective genotyping: (1) nonhost (*L. saligna*) into host (susceptible *L. sativa*) introgression and selection for resistant phenotypes (Fig. 3b), and the other way around: (2) host into nonhost introgression and selection for enhanced infection (Fig. 3c). Strategy 1 was executed at the species level by using nine different *L. saligna* accessions (pictures in Fig. S2) from a broad range of geographic regions including reference accession CGN05271 (Fig. 3a), in order to capture possible genetic variation in genes underlying NHR. Strategy 2 was executed for the reference *L. saligna* CGN05271 accession only.

### Strategy 1: nonhost into host introgression

#### *L. saligna* accessions differ in their degree of NHR

**Fig. 4:**
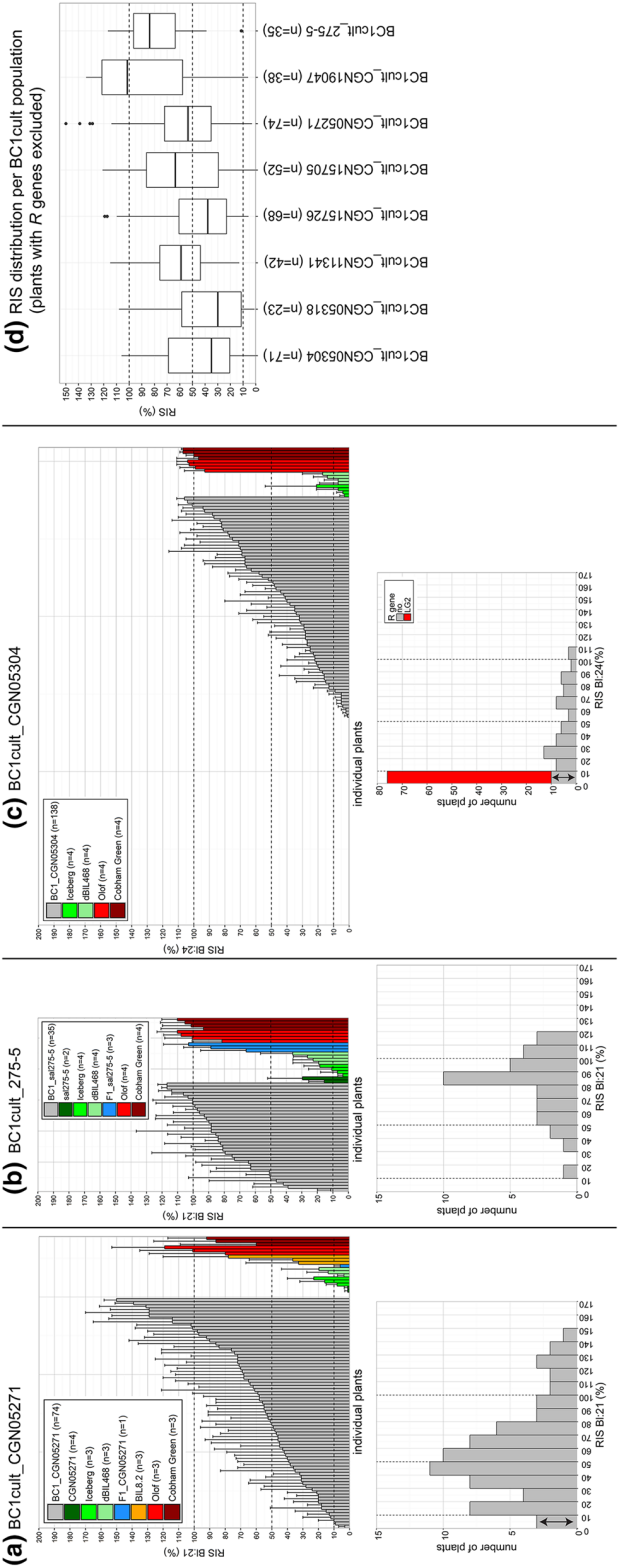
Relative infection severity (RIS) to Bl:21 or Bl:24 of BC1cult populations at adult plant stage (ADTg method) with distinct infection ranges (bar charts) and distribution patterns (histograms). Bar charts include disease test control lines (see legend per population; number of plants tested per line or population indicated in brackets). Bar chart (upper) and corresponding histogram (lower) are shown for **a** BC1cult_CGN05271, **b** BC1cult_CGN19047, and **c** BC1cult_CGN05304; plants in red had a particular marker allele indicating an *R* gene on LG2 against the *B. lactucae* test isolate. Double-headed arrow: plants with RIS ≤ 10% selected for NHR genotyping. **d** RIS boxplots of BC1cult populations. Plants that were highly resistant and explained by qualitative resistance (*R* gene) were excluded. BC1cult_CGN05947 was not included as it had only 13 plants without qualitative resistance. Dashed lines at 10, 50 and 100% RIS are included to ease comparison between figures; 100% RIS: average RIS of susceptible control cv Olof; plants with RIS ≤ 10% were considered as highly resistant

All nine BC1cult populations were tested with Bl:21 or Bl:24. Bl:24 was used for three accessions that were tested for responsiveness to an effector from this isolate (Giesbers et al. 2017). Four BC1cult populations contained plants with hybrid necrosis (HN) symptoms or HN associated allele combinations. These HN plants were discarded (Table S1). All BC1cult populations showed a wide range of infection severity scores. Based on differences in skewness of the distributions between BC1cult populations, we distinguished three types, each represented by one example in Fig. 4 (lower panels with histograms). Bar charts and histograms for all nine BC1cult populations are depicted in Fig. S3–5.

- Distribution type 1 (three BC1cult populations (Fig S3), example: BC1cult_CGN05271 shown in Fig. 4a): Normally or right skewed distributions with a few percent of highly resistant plants (RIS ≤ 10%), indicating segregation of minor genes for quantitative resistance only.
- Distribution type 2 (two BC1cult populations (Fig. S4), example BC1cult_275-5 shown in Fig. 4b): Left skewed distributions with no highly resistant plants (RIS ≤ 10%) (except one plant in BC1_19047) and a low proportion of plants with RIS < 50% compared to type 1 distributions. This distribution indicates segregation of minor genes for quantitative resistance only, with more high average infection scores than in distribution type 1.
- Distribution type 3 (four BC1cult populations (Fig. S5), example BC1cult_CGN05304 shown in Fig. Dc): Extremely right skewed distributions with a very high proportion of highly resistant plants (RIS ≤ 10%). This distribution indicates the presence of qualitative resistance (due to *R* genes), while the remaining plants without *R* genes show a wide and continuous range of infection severity scores, indicating segregation for quantitative resistance. To distinguish the *R* gene based resistant plants from those with quantitative resistance, each resistant plant of the four BC1 populations was checked for co-segregation with markers. In each BC1 population we mapped one (and once two) loci for a major dominant resistance gene (Table S1, Fig. S6). Furthermore, in three of the four populations a proportion of resistant plants did not have *L. saligna* alleles for the markers indicative of the *R* gene. This is illustrated by ten of the 76 resistant plants in BC1cult_CGN05304 (histogram Fig. 4c).

*R* genes in *L. saligna* are accession-specific and race specific (Parra et al. 2016). However, *R* genes seem not essential for NHR as we also observed resistant plants not co-segregating with a wild parent allele at an *R* gene locus. Therefore, BC1cult plants with qualitative resistance due to *R* genes were filtered out to compare population distributions of the remaining plants between *L. saligna* parental accessions (Fig. 4d). It is apparent from this figure that BC1cult_CGN19047 and BC1cult_275-5 have higher average infection severity level compared to other BC1cult populations. Their nonhost–parents were the only *L. saligna* accessions that showed low levels of infection severity at adult plant stage: RIS averaged 5% (CGN19047) or 17% (275-5) in a test with three *B. lactucae* races. Furthermore, their F1s showed infection severity levels to Bl:21 (RIS 73 and 87%) similar to their susceptible *L. sativa* host–parent, while all F1s from the other seven *L. saligna* parents showed low levels of infection severity to Bl:21 (RIS 0, 0, 3, 12, 15, 33 and 47%, Fig. S7). Histological analysis of three *L. saligna* accessions, 275-5, CGN05271 and CGN11341, at young plant stage confirmed that development of *B. lactucae* differs among *L. saligna* accessions (Fig. S8). *L. saligna* 275-5 showed a similar percentage of haustoria (23%) as quantitatively resistant control lines with low infection severities, viz. *L. sativa* lines cv Grand Rapids, cv Iceberg and tripleBIL (description in “Materials and methods” section). *L. saligna* CGN05271 allowed formation of a low proportion of hyphae and haustoria (4%). *L. saligna* CGN11341 only contained a large proportion of malformed hyphae, few normal hyphae and no haustoria.

In three distribution type 1 (right skewed) populations, small proportions (2, 8, and 9%, Table S1) of BC1cult plants were highly resistant. For distribution type 3 (extremely right skewed) populations, a large proportion (17, 48, 62 and 78%, Table S1) of the BC1cult plants was explained by one or two monogenic dominant loci (potential *R* genes). Next to these, fair proportions (13, 14 and 17%, Table S1) of highly resistant plants, without *L. saligna* allele at the potential *R* gene locus, were present in three BC1cult populations of distribution type 3. On average, BC1cult populations with distribution type 1 or 3 harbored 9% highly resistant plants without HN and *R* gene(s) against the test isolate. These highly resistant plants may contain the epistatic alleles underlying NHR.

#### BC1cult selective genotyping

To find the possibly interactive loci underlying high levels of resistance in BC1cult populations, we selected the highly resistant BC1cult plants without HN and *R* gene(s) against the test isolate (criterion: RIS ≤ 10% or occasionally < 15%). This resulted in 32 highly resistant BC1cult plants derived from six *L. saligna* accessions, one to nine plants per population (Table S1). For the progenies of three *L. saligna* accessions, the selection criterion was not met (Table S1). We genotyped the selected plants by 300 genome-wide SNP markers. For these 32 resistant BC1cult plants, we compared the observed genotypic ratio, ‘heterozygous: homozygous host’, with the expected genotypic ratio assuming random inheritance in an unselected population being ‘1:1’. Individual BC1cult subpopulations were too small for statistical analysis to identify significant differences.

**Fig. 5:**
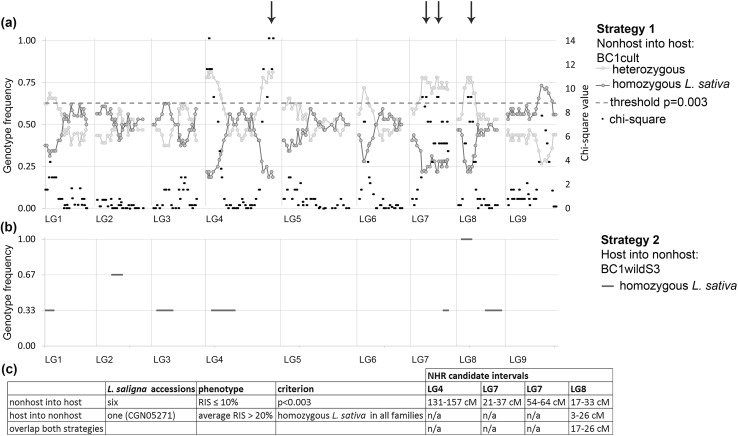
Overview of NHR intervals identified from the “nonhost into host” strategy and from the “host into nonhost” strategy **a** “nonhost into host” strategy:  segregation distortion of 300 SNP markers in 32 highly resistant BC1cult plants. Yellow: heterozygous, blue: homozygous *L. sativa*. *X*-axis: linkage map with white space between linkage groups. Left *y*-axis: genotype frequency, right *y*-axis: Chi-square values (black), dashed line: threshold of *p* = 0.003. Arrows indicate the four loci with significant preference for the heterozygous genotype that were identified as NHR loci (the top of LG4 was excluded because this segregation distortion is likely due to a digenic hybrid incompatibility between *L. saligna* and *L. sativa*), **b** “host into nonhost” strategy:  homozygous *L. sativa* introgressions in BC1wildS3 plants selected for enhanced susceptibility. *X*-axis: linkage map with white space between linkage groups, left *y*-axis: genotype frequency. Blue horizontal lines indicate the frequency of the presence of a homozygous *L. sativa* introgression in all BC1wildS3 plants originating from one (out of three) BC1wild ancestors. A homozygous *L. sativa* introgression with frequency one is considered as a region that nullifies NHR, **c** overview of map intervals underlying NHR based on heterozygous introgressions in BC1cult (*p* < 0.003) and homozygous *L. saligna* introgressions in all BC1wildS3 plants. Centimorgans are based on the F2 consensus map (see “Materials and methods” section) (color figure online)

Chi-square tests of actual versus Mendelian DNA-marker segregation for all 32 plants revealed a preference for *L. saligna* alleles (*p* < 0.003) at five genomic regions (Fig. 5a, Table S5a). Subanalysis of the 32 plants in three subsets of geographic origin (Israel, Southwest Asia, Europe Fig. 3a) or in plants from only five accessions (Table S5a) did not reveal other regions. The five regions were identified on LG4 (two regions), LG7 (two regions), and LG8. However, the overrepresentation of *L. saligna* alleles on the top of LG4 is likely due to transmission ratio distortion (distorted segregation), because such preference for the *L. saligna* allele was observed earlier in two genome-wide genotyped interspecific populations without phenotypic selection: BC1cult_CGN05271 and BC1cult_CGN15705 (Table S5b). We found proof that this distortion in hybrids of CGN05271 is due to a digenic hybrid incompatibility (Den Boer et al. 2014). Therefore, we exclude the top of LG4 as a NHR locus. The four remaining regions that may carry NHR genes (Table S5) carried the *L.* *saligna* allele in 70–80% of the plants (Table S6a). Six combinations of heterozygous introgressions at two, three or four loci were present. On average, between three and four of the four loci were heterozygous in the 32 resistant BC1cult plants (Table 1). Only two plants were detected with just two, and two plants with just one heterozygous introgression(s) out of the four loci. These four plants had *L. saligna* alleles at other loci in the genome, which, in combination, may explain their resistance. Our results suggest that NHR in all accessions is explained by combinations of several genes, in which in particular three or more of the four regions on LG4, LG7 (two regions) and LG8 participate.

**Table 1:**
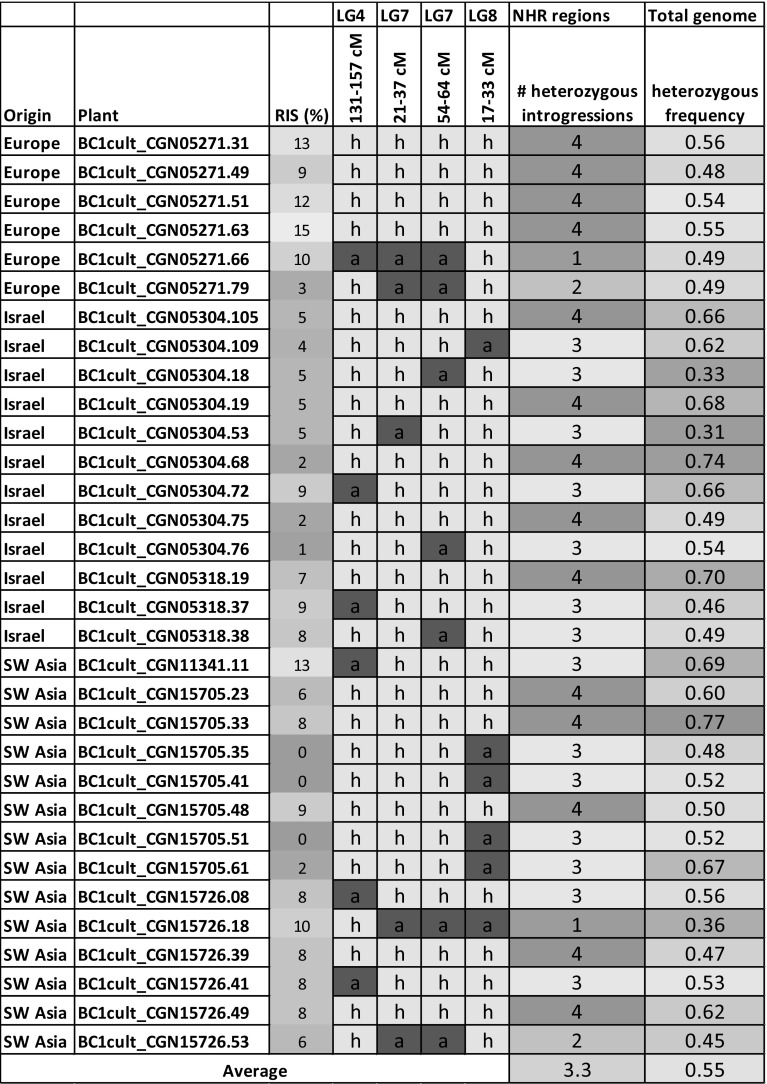
Genotype within the four NHR regions for each individual resistant BC1cult plant (color online)

Genotypes for each interval are represented by the marker(s) with the highest Chi-square value. Genotype codes: a (blue): homozygous *L. sativa*, h (yellow): heterozygous. Numbers are color-formatted with intensities from green (low) to high (red) to facilitate interpretation

*RIS* relative infection severity

### Strategy 2: host into nonhost introgression

As a complementary approach that could reveal evidence for NHR loci, we selected for enhanced infection severity in inbred generations of BC1wild of *L. saligna* CGN05271 (Fig. 3c). The genotypes of BC1wild plants are on average half ‘homozygous nonhost’ and half ‘heterozygous’. BC1wild plants were inbred for three generations. The frequency of the ‘homozygous host (*L. sativa*)’-genotype is increasing in every next inbred generation. In the third inbred generation (BC1wildS3), the expected genotype frequencies will be, in the absence of selection: 0.72 homozygous nonhost (*L. saligna*), 0.06 heterozygous and 0.22 homozygous host (*L. sativa*) (Fig. 3c). Repeatedly inbreeding increases the rate of homozygous host regions, some of which may be associated with enhanced levels of susceptibility (absence of certain NHR alleles). In each generation, we therefore selected plants with sporulation (BC1wildS1: RIS > 0%, BC1wildS2: RIS > 10%) as the ancestors of the subsequent inbred population. Hundreds of plants were disease phenotyped over the three inbred generations (Fig. S9).

As shown in Fig. 2a all BC1wild plants displayed a very low infection severity level (mean RIS 1%) which indicated that increased infection can only be achieved by homozygous *L. sativa* (host) introgressions. Twenty-eight BC1wild plants were inbred resulting in 26 BC1wildS1 families (two BC1wild plants did not produce offspring), with theoretically expected genotype frequencies of 0.63 homozygous *L. saligna*, 0.25 heterozygous and 0.13 homozygous *L. sativa*. The complete genome of the host–parent *L. sativa* cv Olof was covered by the population of 26 BC1wild plants. Ten BC1wild plants were enough to cover each locus at least once (heterozygous introgressions in ten BC1wild plants, Table S6b). We tested 26 BC1wildS1 families (average: 20 plants/family) at young plant stage (method YDT), for enhanced susceptibility, observed as sparse sporulation symptoms. In total, 18 plants from seven families showed slight levels of sporulation (1–9% RIS). These 18 plants were retested in an ADTg and showed RIS levels of 0–10% (Fig. S10).

Sixteen BC1wildS1 plants showing sporulation in the YDT and/or ADTg were selected for further inbreeding and resulted in only five BC1wildS2 families from three different BC1wild ancestors. Eleven BC1wildS1 plants did not produce offspring due to low vitality and/or low fertility. The germination frequency of BC1wildS2 families was severely reduced and ranged from 0.12 to 0.60, with an average of 0.36 for all families together. On average, six plants per BC1S2 family were disease phenotyped (Fig. S11). Eight BC1wildS2 plants with RIS > 10% were selected for further inbreeding, resulting in only five BC1wildS3 families from three different BC1wild ancestors. Three BC1wildS2 plants did not produce offspring due to low vitality and/or fertility.

The five resulting BC1wildS3 families (17–20 plants/family) were genotyped and phenotyped. They contained five to nine regions with host introgressions and had average genotype frequencies for the “homozygous host’-genotype below or close to the expected 0.22 (range 0.05–0.21, Table S6). The five BC1wildS3 families (17–20 plants/family) showed increased levels of infection severity compared to the original *L. saligna* CGN05271 parent (Fig. S12). Their infection severity levels ranged from similar to control lines with ‘high level of quantitative resistance’ (cv Iceberg and dBIL468) to intermediate levels (in between levels of cv Iceberg and BIL8.2). Disease test validation at seedling stage in subsets of BC1S4 lines per BC1S3 family showed similar infection levels as at adult plant stage (Fig. S13). These five BC1wildS3 families originated from three BC1wild individuals, in which 90% of the genome from the susceptible host–parent *L. sativa* cv. Olof was covered.

All BC1wildS3 plants (from five families) contained the same introgressed region with a homozygous *L. sativa* introgression on LG8 (3.4–26.4 cM); this region overlapped with the locus on LG8 identified in resistant BC1cult plants (Fig. 5). Other commonly found homozygous *L. sativa* introgressions were present in maximally three out of five families and did not overlap with NHR regions identified in the resistant BC1cult plants (Fig. 5). We noticed that five out of 13 (38%) segregating introgression segments in the BC1wildS3 plants showed distorted genotype frequencies with a lack of the homozygous host genotype (Table S6). Possibly this decreased the chance for introgression and detection of host segments that nullify NHR.

Three BC1wildS1 plants with sporulation levels of 3–30% RIS were also backcrossed to the host parent *L. sativa* and the 24 cross-products were included in adult plant disease tests along BC1wildS2 and F1-plants from *L. saligna* *× L. sativa *cross. The code for the cross product is ‘BC1wildS1BC1cult', and the expected genotypic composition is 0.75 heterozygous and 0.25 homozygous *L. sativa*. Disease test analysis showed that 3 out of 24 plants had much higher RIS levels than its F1 and BC1wildS1 ancestor (RIS > 80%, Fig. S14) and these three plants were subsequently genotyped. As the F1 generation with a fully heterozygous genotype was highly resistant (low RIS, Fig. 2 and Fig. S14), the high RIS levels in these three BC1wildS1BC1cult plants are expected to be due to certain homozygous *L. sativa *introgressions. Therefore, these loci with homozygous *L. sativa* introgressions are promising loci for NHR genes in *L. saligna*. Apart from a homozygous *L. sativa* introgression on LG8 (which was already fixed in the two BC1wildS1-ancestors), two of the three genotyped BC1wildS1BC1cult plants harbored a homozygous *L. sativa* segment on LG5 (26–40 cM) and LG9 (22–51 cM), that was heterozygous in their BC1wildS1 ancestor. One BC1wildS1BC1cult plant with RIS 100% had a relatively low percentage of homozygous host genome, genotypic composition: 0.08 homozygous *L. sativa* (host), 0.92 heterozygous. This plant harbored a homozygous *L. sativa* introgression on LG3 (21–63 cM) and LG4 (133–157 cM) that was heterozygous in its BC1wildS1 ancestor (Table S6c). The latter region on LG4 falls within the region on LG4 identified from BC1cult plants (strategy 1, nonhost into host introgression).

Summarizing all results, four NHR regions with an overrepresentation of *L. saligna* (nonhost) alleles were identified in highly resistant BC1cult plants from six *L. saligna* accessions. One of these loci, on LG8, overlapped with a locus identified as a homozygous *L. sativa* (host) introgression in all BC1wildS3 lineages from CGN05271 selected for enhanced infection severity. Based on the BC1wildS3 lineages, the position of the putative gene for NHR on LG8 was narrowed down to an 9 cM interval between 17–26 cM (Fig. 5c).

## Discussion

Our goal was to identify the genetic basis of nonhost resistance (NHR) in *L. saligna* at the species level. We identified four chromosomal regions containing a putative set of epistatic genes for NHR through a bidirectional backcross approach.

### NHR status of *L. saligna*

Our analysis of seedling disease test data from the Dutch Centre for Genetic Resources (CGN) demonstrated that *L. saligna* as a species is highly resistant to *B. lactucae* and confirms the results of a less elaborate dataset (van Treuren et al. 2011). The observation of some not fully resistant *L. saligna*–*B. lactucae* interactions at seedling stage (Fig. S2) may be a plant stage-dependent effect. Bonnier et al. (1992) and Petrželová et al. (2011) reported occasional sparse sporulation on *L. saligna* at seedling stage, but full resistance at adult plant stage. Petrželová et al. (2011) identified accession 275-5 as one of the lines with the highest level of infection severity, with 24% sporulation at seedling stage but no sporulation at adult plant stage. We validated the taxonomic classification of this accession. In our experiments, its observed infection severity to *B. lactucae* was 72% RIS at seedling stage and even 17% RIS at adult plant stage. In conclusion, we confirm the nonhost status of *L. saligna*, despite the exception of this one accession.

### NHR is based on resistance factors from *L. saligna*

Comparing F1, BC1wild and BC1cult of CGN05271 showed, respectively, a low infection, overall low infection and a continuous range of infection severity levels. If dominant susceptibility genes of host *L. sativa* were involved, all BC1cult plants should have been susceptible, as well as the F1 and part of the BC1wild. The low percentage of highly resistant BC1cult plants and the high resistance levels of the F1 plants indicated that multiple heterozygous introgressions, each with one copy of the nonhost allele, can lead to high levels of resistance. The few highly resistant BC1cult plants most probably harbor a combination of particular heterozygous introgressions leading to resistance.

### The degree of NHR differs between accessions

To study NHR at the species level, we extended our study to eight additional *L. saligna* accessions from a wide range of geographic origins. Variation for resistance genes is not expected in a nonhost, but may be incidentally present (Antonovics et al. 2012); therefore, we expected to identify NHR loci in common within the nine accessions. Infection severity levels in F1 plants and BC1cult populations derived from different *L. saligna* accessions showed that the genetic dose of NHR differs between *L. saligna* accessions. *L. saligna* CGN19047 and 275-5 had a higher RIS average for F1 and BC1cult populations than the other *L. saligna* accessions. Furthermore, *L. saligna* 275-5 itself showed an average RIS of 17% at adult plant stage and in histological analysis it had a similar proportion of haustoria as in quantitatively resistant control lines, whereas the other tested *L. saligna* accessions showed very few or no haustoria. Accession CGN19047 and 275-5 may lack the same NHR locus allele(s) by descent as they are genetically most similar and collected from geographically close regions among the nine accessions (Fig. 3a). If the remaining NHR genes of these accessions would lose function due to incidental mutations, *L. saligna* might eventually evolve into a novel host species for *B. lactucae*. Crossings between accessions with the lowest dose of NHR (275-5 and CGN19047) and completely resistant *L. saligna* accessions could lead to identification of the gene(s) that lowered the dosage of NHR.

### Confounding factors

Two factors complicated our study of NHR in certain crosses of *L. saligna* accessions with *L. sativa*: (1) a hybrid incompatibility symptom, hybrid necrosis (HN), visible as necrotic flecks on leaves in four of the nine BC1cult populations. Jeuken et al. (2009) demonstrated that such an HN reaction is associated with resistance to *B. lactucae.* (2) The segregation of monogenic dominant resistances (qualitative resistance, *R* genes) in four other BC1cult populations.

The presence of *R* genes as well as genes for quantitative resistance in *L. saligna* seems in line with the presumed combined action of *R* genes and pattern recognition receptors (PRRs) in NHR as proposed by the model of Schulze-Lefert and Panstruga (2011). However, we showed that *R* genes are not essential for NHR, as five out of nine *L. saligna* accessions did not contain an *R* gene against the test isolate, but were completely resistant. Furthermore, in all accessions QTL mediated resistance was present that led to high or intermediate resistance levels in BC1cult populations.

Possibly, the occasional *R* genes are remnants of an ancient host status of *L. saligna*. *B. lactucae* may later have specialized on *L. serriola,* the probable ancestor of *L. sativa* (De Vries 1997; Zhang et al. 2017). Alternatively or additionally, accession-specific *R* genes may be an accidental by-product of gene duplications, recombination, unequal crossing-over, point mutations and diversifying selection that are common within *R* gene clusters (Meyers et al. 2005). Sequence exchange between *R* genes that are effective against a specific pathogen can even result in the generation of novel *R* genes with resistance specificities to other, phylogenetically unrelated pathogens (Slootweg et al. 2017). This flexibility of *R* genes to generate novel resistance specificities may have resulted in coincidental presence of *R* genes in the *L. saligna* nonhost. Another point to be addressed here is that the presence of *R* genes to a nonadapted pathogen in a nonhost remains unnoticed as long as nonhosts are not interfertile with hosts, which is commonplace with the exception of our interspecific cross. Possibly the frequency of *R* genes that are functional against nonpathogens in nonhosts is higher than we are aware of and we generally expect.

### Identification of four potential components for NHR

Two to 17% of the BC1cult plants per population were highly resistant (≤ 10% RIS; after exclusion of HN and *R* gene explained plants), resulting in 32 BC1cult plants of six *L. saligna* accessions. If the high level of resistance would be explained by a particular combination of a certain number of loci, it would imply a set of three to five underlying genes (½^3^ = 0.125, ½^4^ = 0.0625, ½^5^ = 0.0313). In NHR studies of *Arabidopsis* mutants, minimal two (*pen*3 + *eds*1) and three (*pen*2 + *pad*4 + *sag*101) mutated genes were needed to lead to dense sporulation of pea powdery mildew and occasional sporulation of barley powdery mildew (*Bgh*) (Lipka et al. 2005; Stein et al. 2006).

Our strict phenotypic selection of highly resistant BC1cult plants (RIS ≤ 10%) and subsequent strict genotypic selection (overrepresentation of *L. saligna* alleles with *p* < 0.003) resulted in the identification of four NHR loci. These loci were located on LG4, LG7 (two regions) and LG8. In former NHR genetic studies on accession CGN05271, we detected, among others, individual QTLs at three similar locations, but these were effective only in certain plant developmental stages: *rbq15* (LG4), *rbq6* (LG8) and *rbq1* (LG7) (Jeuken and Lindhout 2002; Zhang et al. 2009a). The fact that the remaining 12 QTLs that were identified earlier were not detected this time may suggest that these QTLs are not essential for NHR, or not in all accessions.

The high resistance level of the selected BC1cult plants may be explained by five combinations of heterozygous segments at three or four of the identified NHR loci. Therefore, resistance alleles at minimal three loci seem necessary for NHR of *L. saligna*. If all five possible combinations of at least three loci (combinations in binary code: 1110, 1101, 1011, 0111, 1111) would lead to high resistance, the probability of resistant plants in a BC1cult would be 31% (5 out of 16 possible combinations of 4 loci = 0.315, if under Mendelian segregation). However, we found on average 9% highly resistant BC1 plants. We have indications that the NHR loci are closely linked to regions with distorted segregation with a bias toward host (*L. sativa*) alleles. This could explain our lower proportion of resistant plants. In these 32 BC1 genotypes, we could not identify a particularly common combination out of these five combinations. Possibly, few other loci together with three of the four detected loci might explain NHR. In three  enhanced susceptible (RIS > 80%) plants with an expected genotypic composition of 0.75 heterozygosity and 0.25 homozygous *L. sativa *(BC1wildS1BC1cult), we identified other possible NHR loci on LG3, LG5 and LG9 and validated the NHR locus on LG4.

Individual BC1cult populations were too small to study whether there was significant genetic variance for NHR between accessions, as 32% of all 615 tested BC1cult plants had to be discarded due to the presence of confounding HN or *R* genes. To obtain a larger dataset, genotyping resistant BC1cult plants of *L. saligna* CGN15726 with Bl:21 would be most efficient, as these plants did not show HN and no *R* genes against Bl:21. The previously identified QTLs in BILs were race-nonspecific (Zhang et al. 2009a). Testing with multiple isolates would be necessary to prove race-nonspecificity of the identified NHR loci. Furthermore, field tests should be conducted to exclude plant-stage-dependent effects of NHR loci, as previously found for some partially resistant BILs (Zhang et al. 2009a).

### Additional evidence for a NHR component on LG8

Through introgression of host segments into a nonhost genetic background (CGN05271), we identified one host (*L. sativa*) derived introgression region leading to enhanced infection in all five BC1wildS3 lineages (with low to intermediate sporulation). This region overlapped with the NHR region on LG8 identified in resistant BC1cult plants with an interval from 17 to 26 cM (spanning 15 Mb). The host into nonhost strategy did not convincingly confirm the other detected NHR loci on LG4 and LG7 nor did it identify a new locus. This might partly be explained by chance, due to relatively low numbers (average *n* = 6 per ancestor) in the second inbred generation, BC1wildS2. Additionally, it might be explained by the distorted segregations at five of the thirteen segregating introgression segments, which showed distorted genotype frequencies with a lack of the homozygous host genotype.

## Conclusion and recommendations for further research

Our findings indicated that: the genetic dose of NHR differs between *L. saligna* accessions and NHR in *L. saligna* seems explained by combinations of epistatic genes on three or four chromosome segments (Fig. 5c). Validation and fine mapping of the four identified NHR loci in next inbred generations will ultimately lead to the goal of cloning NHR genes.

### Author contribution statement

AG and MJ designed the research and wrote the manuscript. AG performed the genotype–phenotype experiments with contributions of EdB (two populations), DB (one population) and TB (one population). JS performed the histology experiment. AG analyzed all experiments. MvK performed SNP calling. RN and RV were involved in revising the manuscript critically.

## Electronic supplementary material

Below is the link to the electronic supplementary material.

Supplementary material 1 (XLSX 239 kb)

Supplementary material 2 (DOCX 9323 kb)
