# Supplementary material for: Bidirectional backcrosses between wild and cultivated lettuce identify loci involved in nonhost resistance to downy mildew
Source: Theor Appl Genet. 2018 May 25;131(8):1761–76. doi: 10.1007/s00122-018-3112-8 (PMC6061147; doi:10.1007/s00122-018-3112-8)
Supplement: Supplementary file 2 — Supplementary material 2 (DOCX 9323 kb) [file 122_2018_3112_MOESM2_ESM.docx]

**Supplemental Figures**


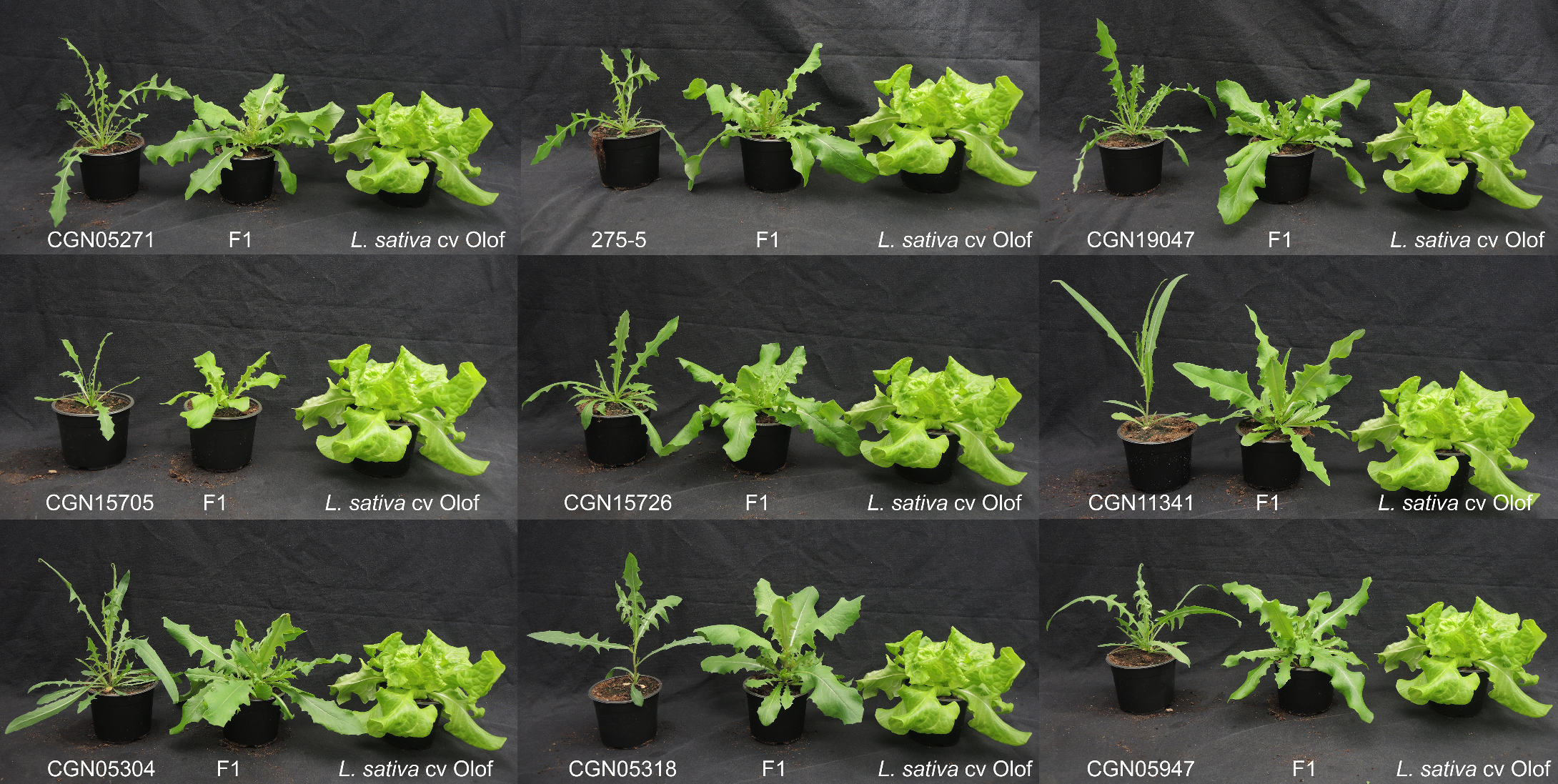


**Figure S1. Phenotypes of all tested *L. saligna* accessions, the F1 hybrid and *L. sativa* cv Olof at five weeks of age.**


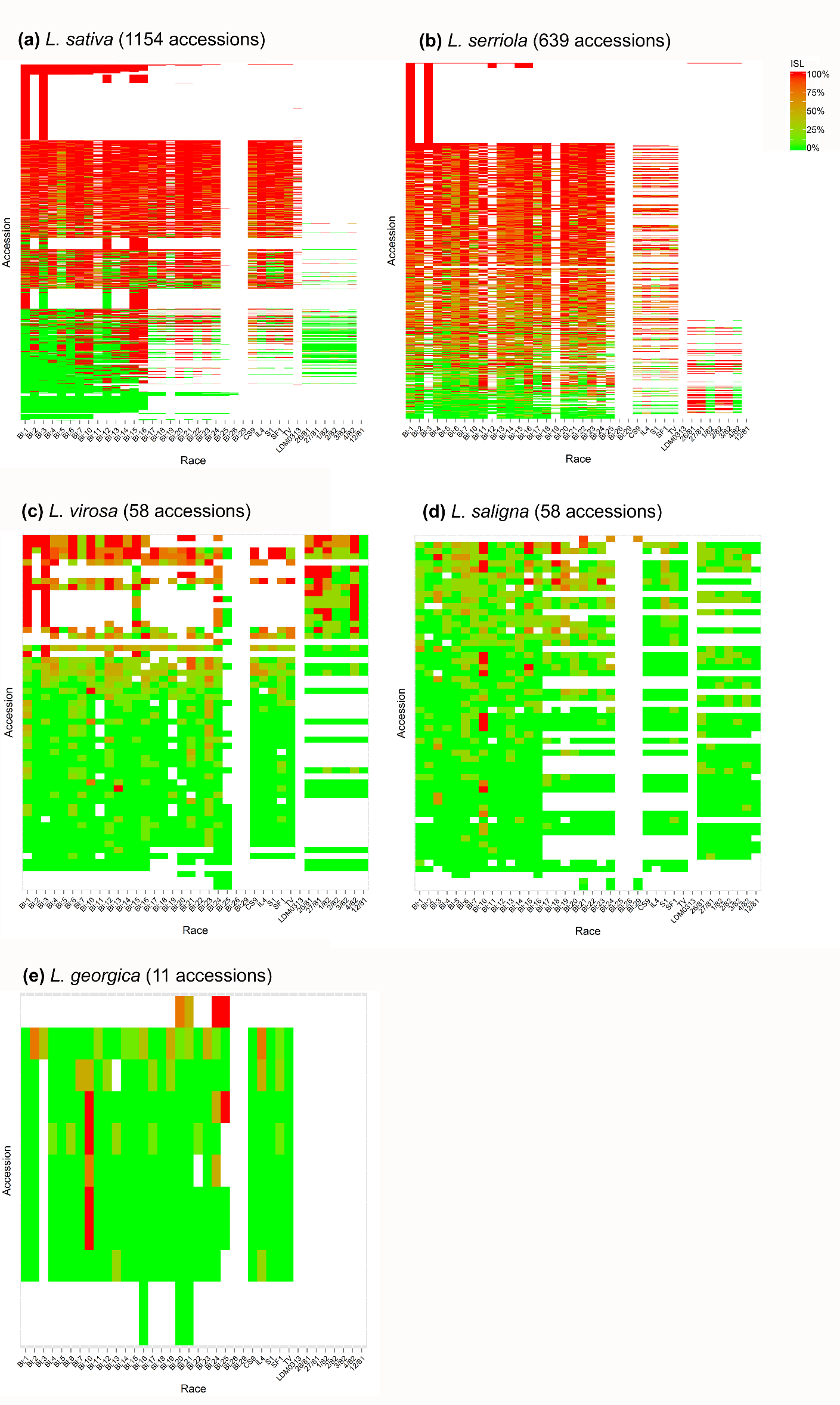


**Figure S2. Heatmap of seedling infection severity level (ISL) of all individual interactions between each *Lactuca* accession and *B. lactucae* race.** **(a)** *L. sativa* **(b)** *L. serriola* **(c)** *L. virosa* **(d)** *L. saligna*. Resistant (0%: green) to susceptible (100%: red) of all tested accessions to each race, per Lactuca species. No colour (white) means that a certain accession x *B. lactucae* race combination has not been tested.

Data are derived from <http://cgn.websites.wur.nl/Website/downloads/DownloadCnr06.htm> and supplemented with our own data on three L. saligna accessions.

**
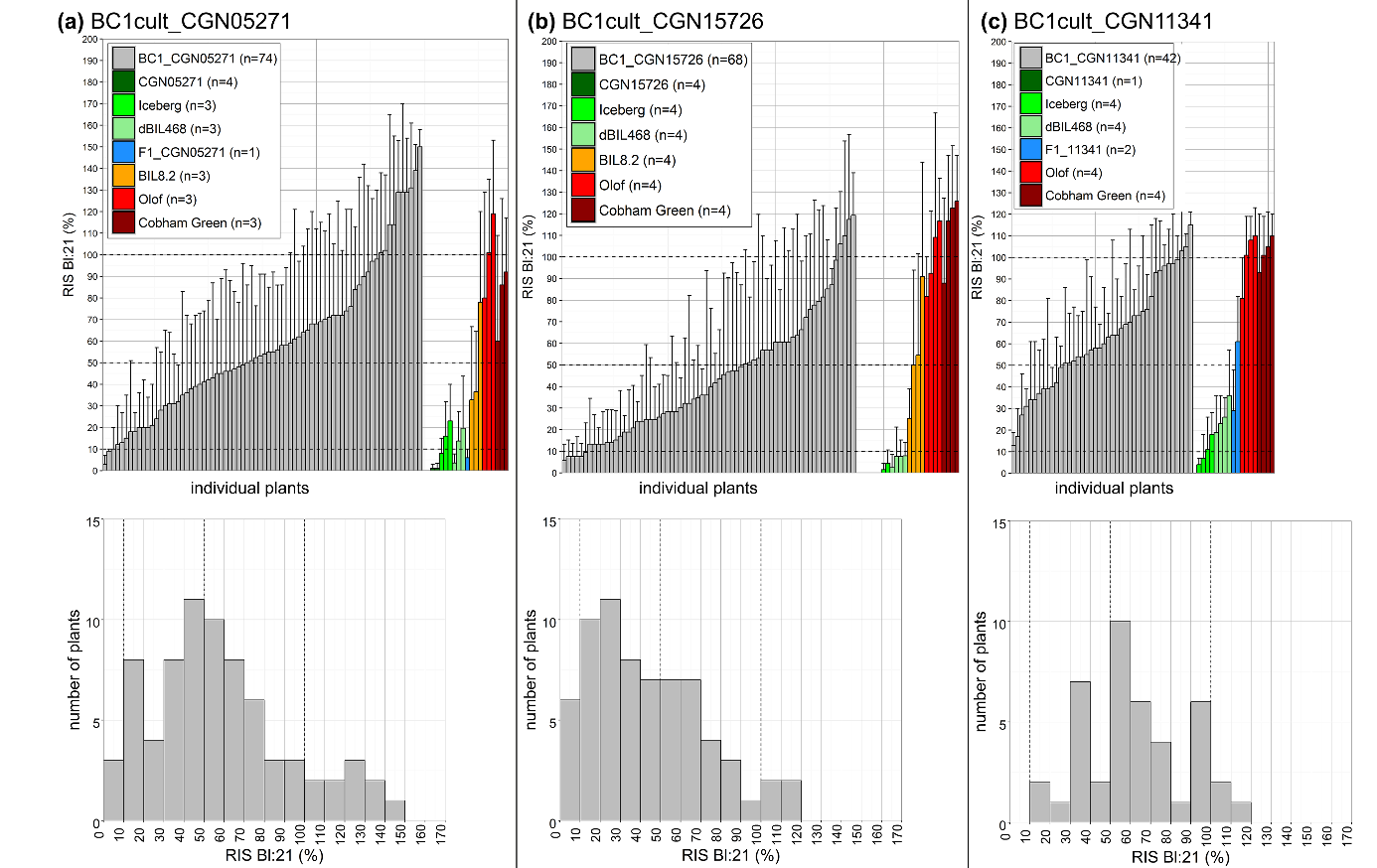
**

**Figure S3. Bar charts including disease test controls (see legend) and corresponding histograms with normally or right skewed distributions (type 1) of relative infection severity levels (RIS) of three BC1cult populations at adult plant stage without a major resistance (R) gene against the test isolate** **(a)** BC1cult_CGN05271 **(b)** BC1cult_CGN15726 **(c)** BC1cult_CGN11341. Double-headed arrow: plants with RIS ≤10% selected for NHR genotyping.

**
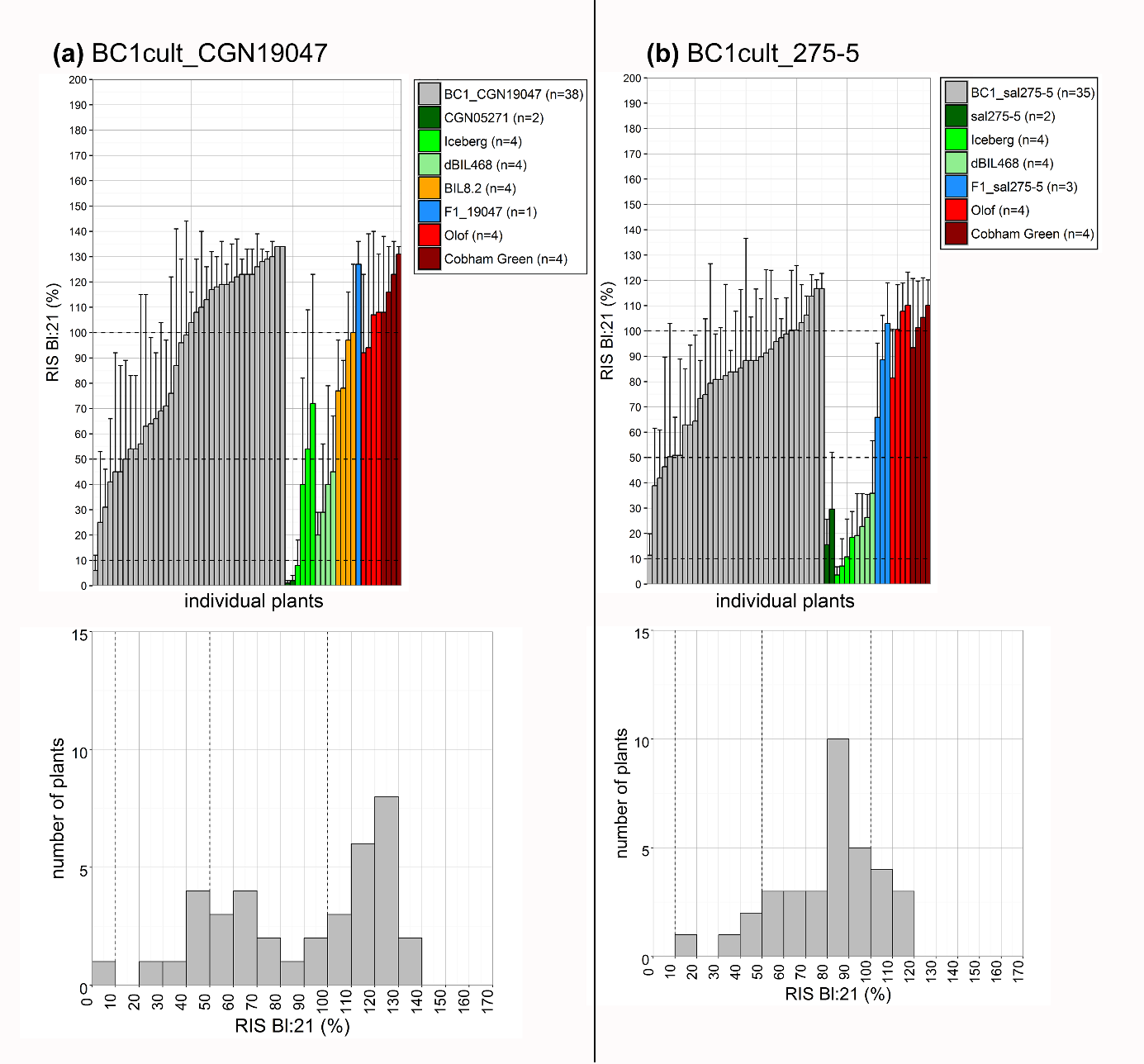
**

**Figure S4. Bar charts including disease test controls (see legend) and corresponding histograms with left skewed distributions (type 2) of relative infection severity levels (RIS) of two BC1cult populations at adult plant stage without a major resistance (R) gene against the test isolate (a)** BC1cult_CGN19047 **(b)** BC1cult_275-5.

**
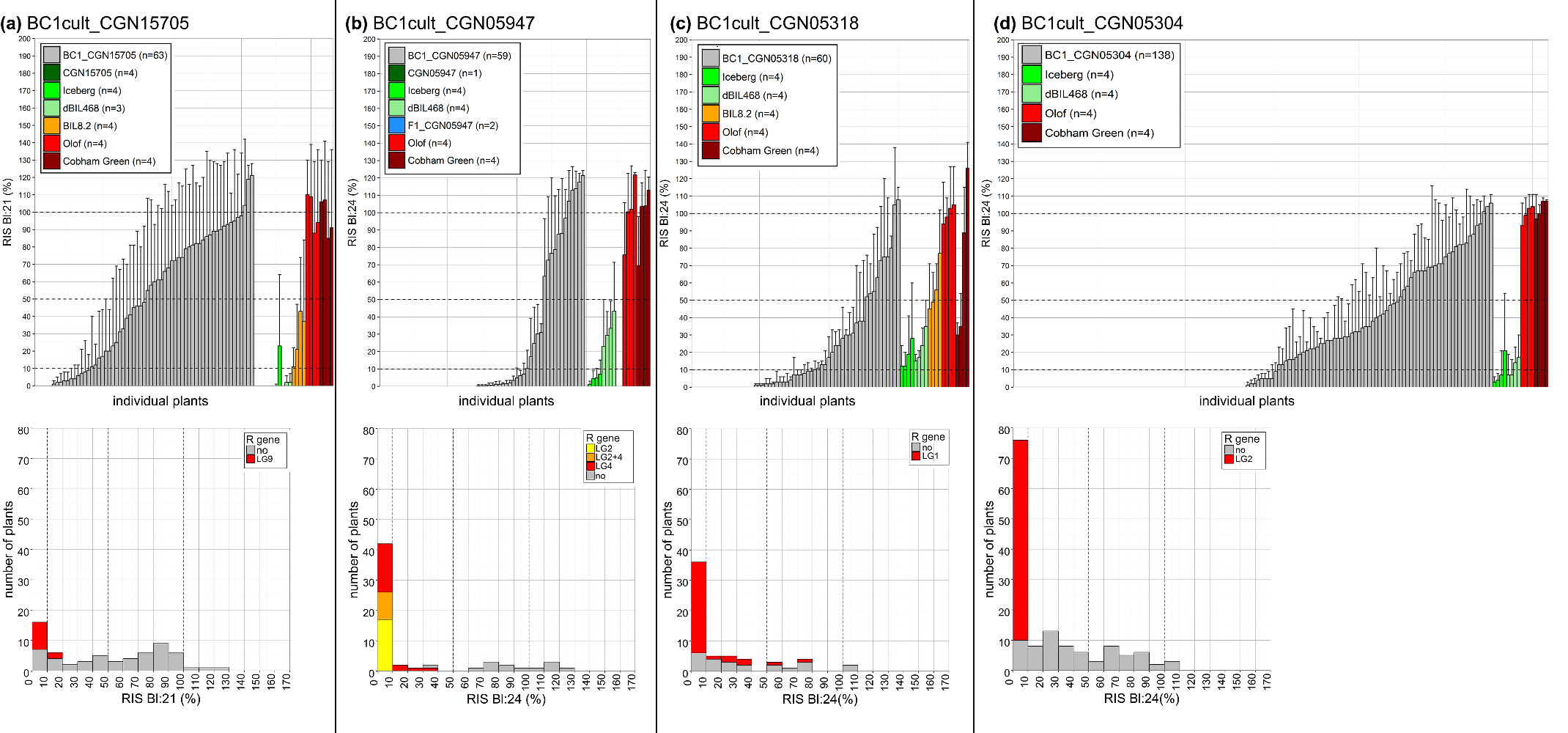
**

**Figure S5. Bar charts including disease test controls (see legend) and corresponding histograms with extremely right skewed distributions (type 3) of relative infection severity levels (RIS) of four BC1cult populations at adult plant stage with a major resistance (R) gene against the test isolate (a)** BC1cult_CGN15705 **(b)** BC1cult_CGN05947 **(c)** BC1cult_CGN05318 **(d)** BC1cult_CGN05304. In the histograms, plants in red and yellow showed co-segregation between a single dominant L. saligna allele (R gene) and resistance against the B. lactucae test isolate on LG1, LG2, LG4 or LG9 as indicated in the legend. Double-headed arrow: plants with RIS ≤10% selected for NHR genotyping

**
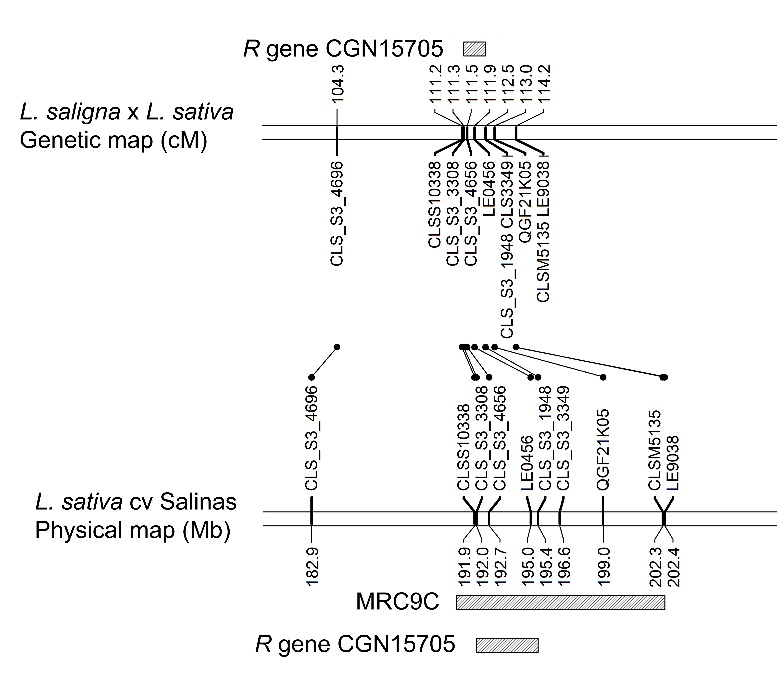
**

**Figure S6. Map interval of the *R* gene on linkage group 9 in *Lactuca saligna* CGN15705.** Major resistance cluster 9C (MRC9C) in *Lactuca sativa* cv. Salinas is shown below the physical map. The genetic map distances are based on an F2 population of *L. saligna* CGN05271 x *L. sativa* cv. Olof. The physical map distances are from *Lactuca sativa* cv Salinas genome assembly v8 (http://lgr.genomecenter.ucdavis.edu/, Reyes-Chin-Wo *et al.* 2017).


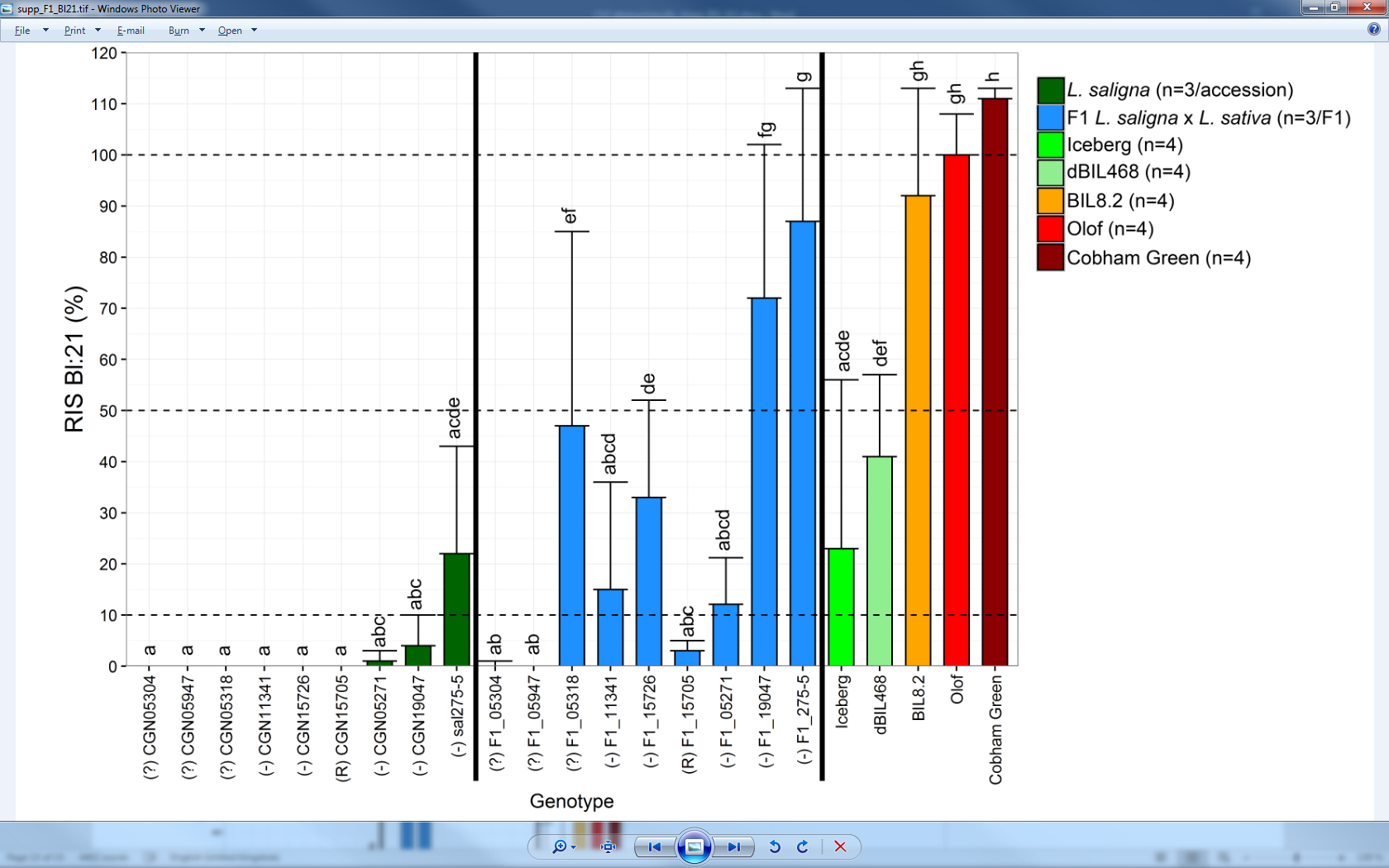


**Figure S7. Relative infection severity (RIS) to B. lactucae Bl:21of L. saligna parental accessions, F1-genotypes and disease test control lines at adult plant stage.** For ease of interpretation, the three groups of plants are separated by black bars. Letters in common indicate no significant difference (α =0.05, Bonferroni test). Per genotype three plants were evaluated with 2-4 leaf parts per plant. Prefix to test lines (-) no R gene identified against the test isolate, (R) R gene identified against the test isolate (by RIS segregation in BC1cult population and molecular marker analysis), (?) presence of R gene against test isolate unknown, because the corresponding BC1cult population was not tested with this test isolate.

**Figure S8. Graphical representation of the infection process of B. lactucae race Bl:21 in tested Lactuca genotypes 48 hours post inoculation.** The infection structures, which B. lactucae may develop while invading lettuce plants, are represented on the left side with differently coloured arrows: PV= primary vesicle, SV= secondary vesicle, MAL-HY= malformed hypha, HY= hypha and HA= complete infection unit with haustoria. The width of an arrow indicates relatively how many infection units reached a developmental stage. The width of the arrows together represents per genotype 100% of the infection units. Values mentioned below the name of each accession/line represent respectively the infection severity level (%) of the macroscopic controls at 10 dpi and the total number of observed infection units (IU).


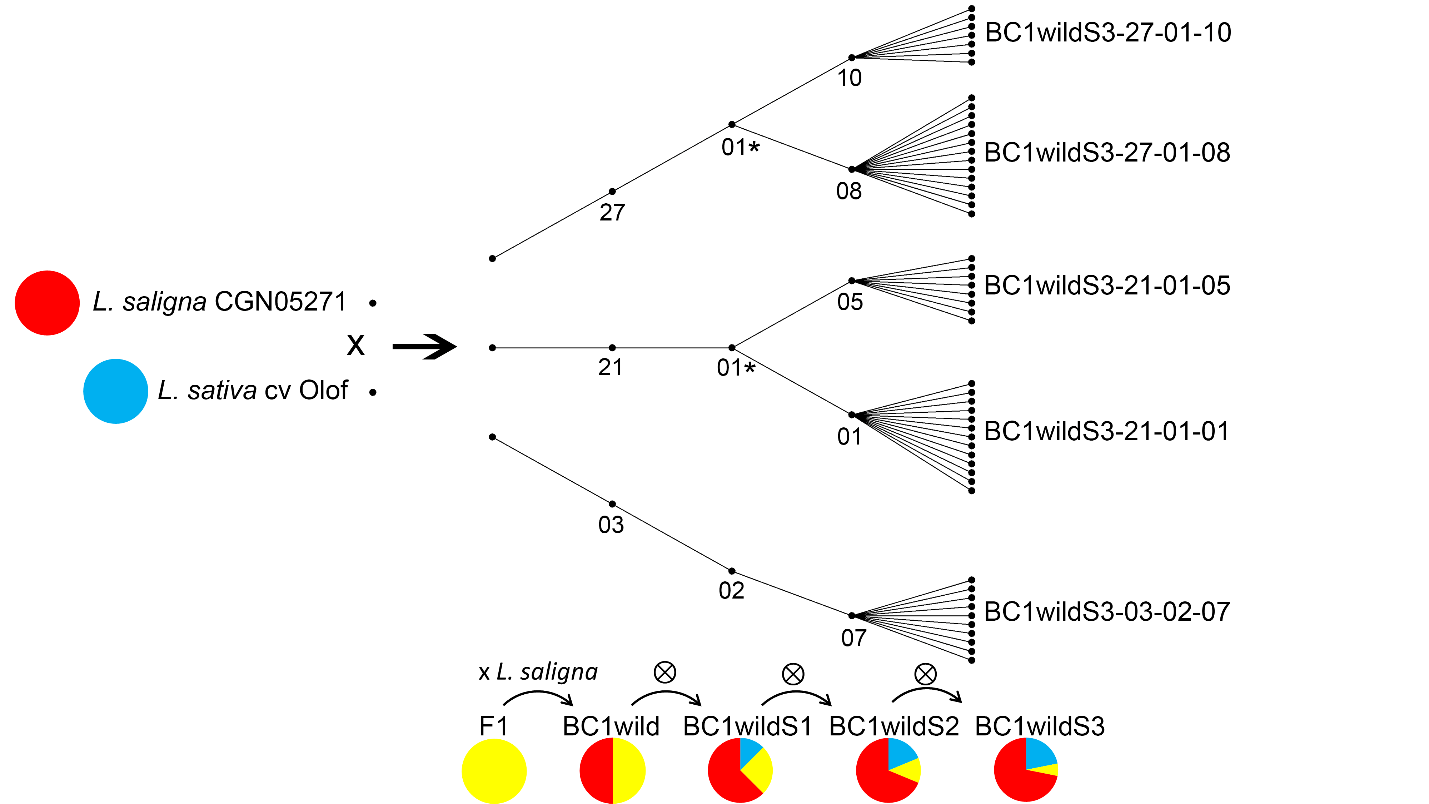

**Figure S9. Pedigree of genotyped BC1wildS3 lineages and overview of phenotyped and genotyped plants.** * Backcrossed to L. sativa, resulting in BC1wildS1BC1cult families (Fig. S14). Pie charts depict the average genotypic composition of each generation. Red: homozygous L. saligna, blue: homozygous L. sativa, yellow: heterozygous. The table lists details on plant numbers that were phenotyped and genotyped. Not all selected plants resulted in a successful next inbred generation, due to reduced vitality and fertility. RIS: relative infection severity, n/a: not applicable.


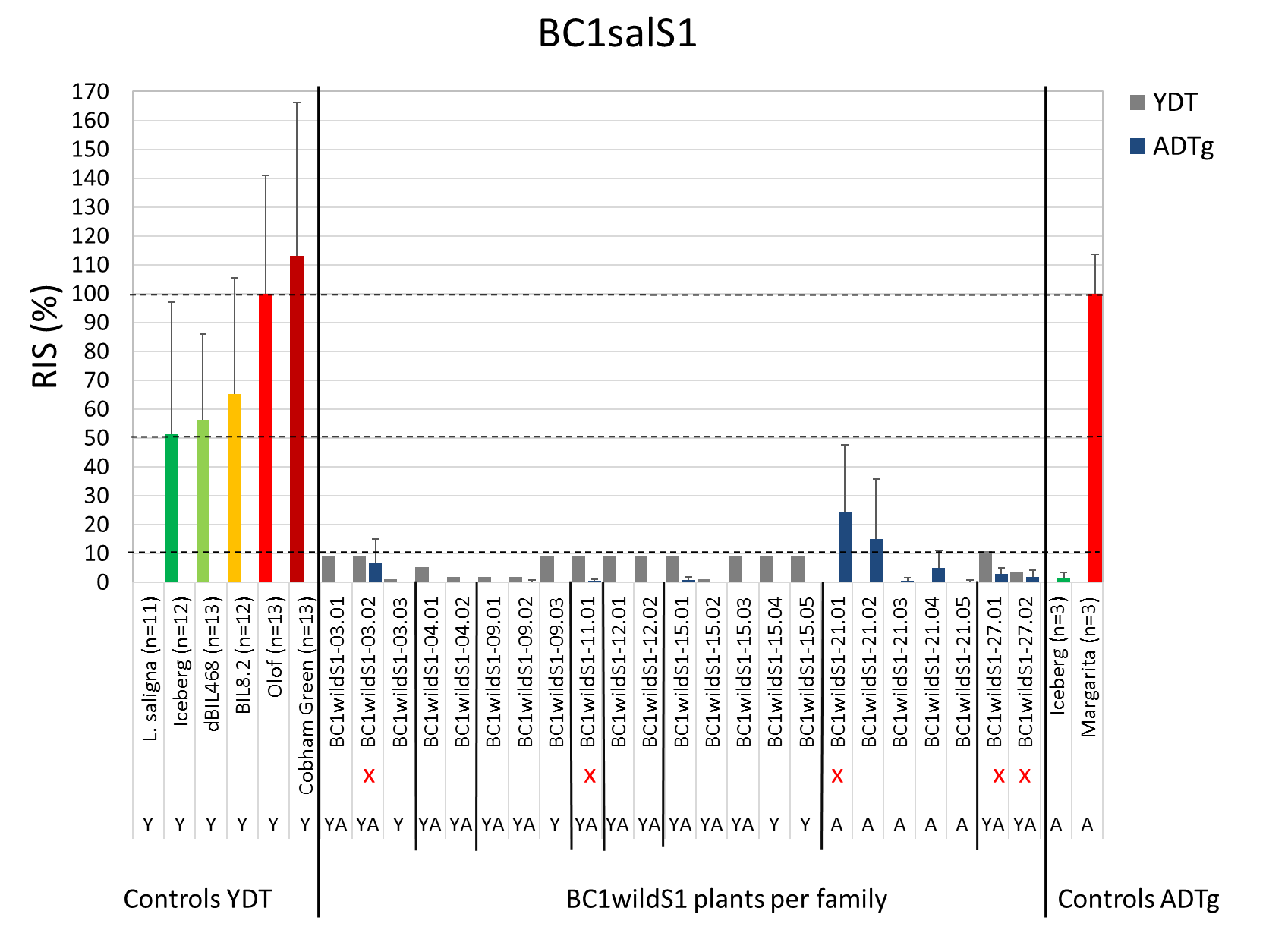


**Figure S10. Relative infection severity (RIS) to Bl:21 of BC1wildS1 plants in a young plant disease test (YDT) and adult plant disease test (ADTg).** Plants from lineage BC1wildS1-21 were included in the ADTg as they escaped proper disease test conditions due to local inferior humidity conditions in the YDT. YDT controls are depicted on the left, ADTg controls on the right. Results of BC1wildS1 plants are depicted in the middle, with different families separated by vertical lines. Y: tested in YDT, A: tested in ADTg. Red crosses indicate plants selected as mothers for BC1wildS2 families.


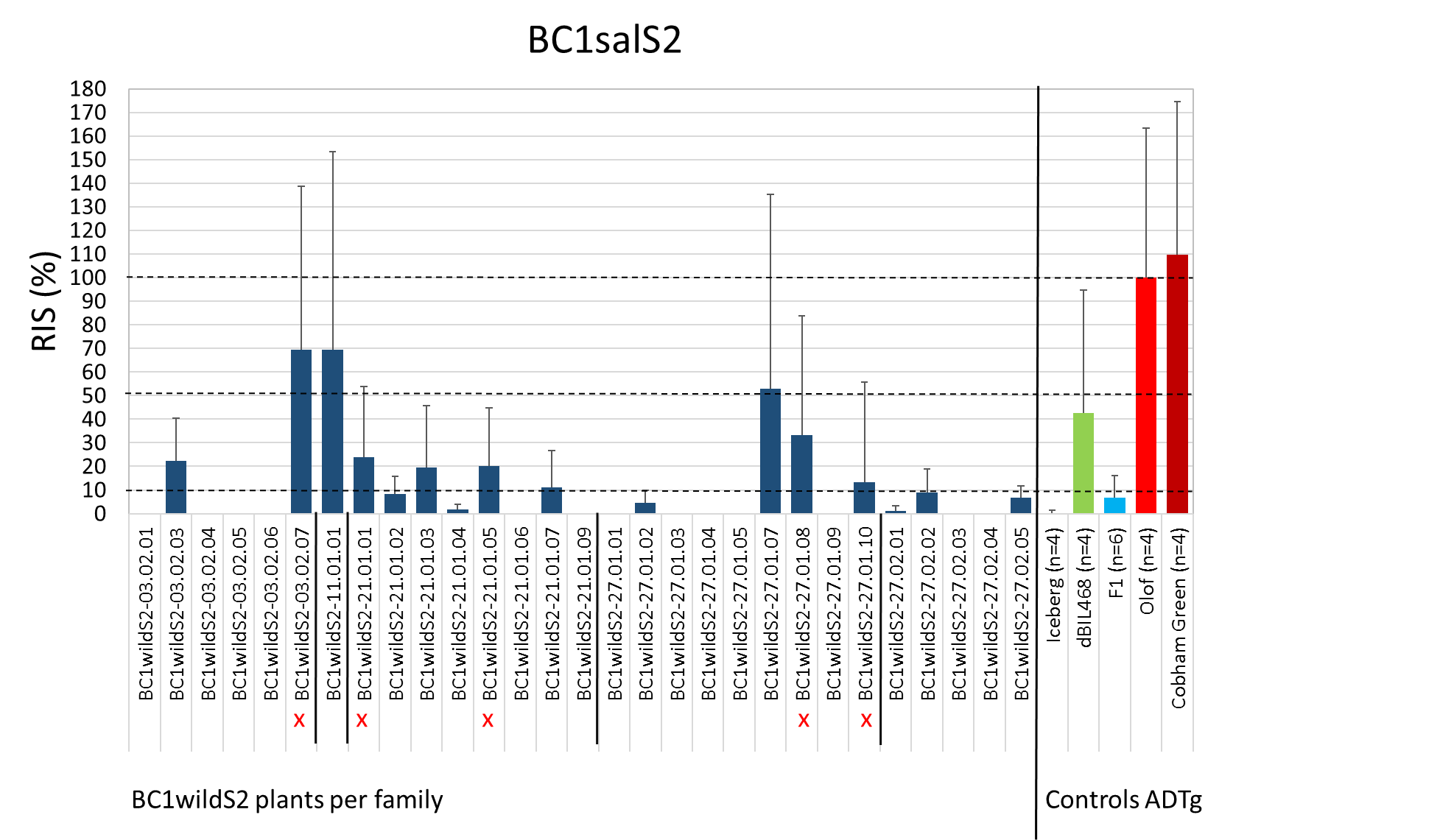


**Figure S11. Relative infection severity (RIS) to Bl:21 of BC1wildS2 families with BC1wildS1 mothers with enhanced susceptibility and control lines in an adult plant disease test (ADTg).** Relative infection severity levels of BC1wildS2 families are separated by vertical lines. Red crosses indicate plants serving as mothers for BC1wildS3 families.


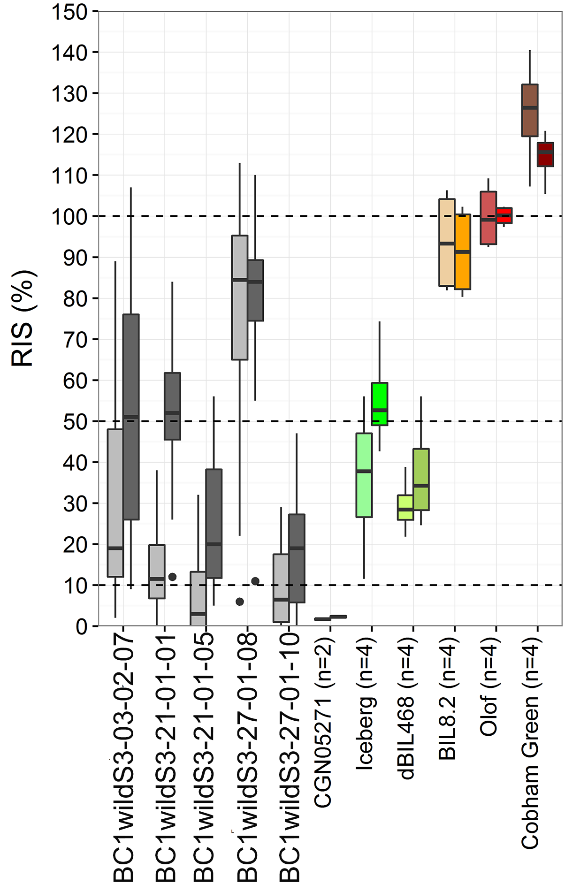


**Figure S12. Relative infection severity (RIS) to Bl:21 of BC1wildS3 families selected for enhanced susceptibility and controls.** Per population type, RIS at 9 dpi is plotted in a light colour followed by RIS at 12 dpi in a darker colour.


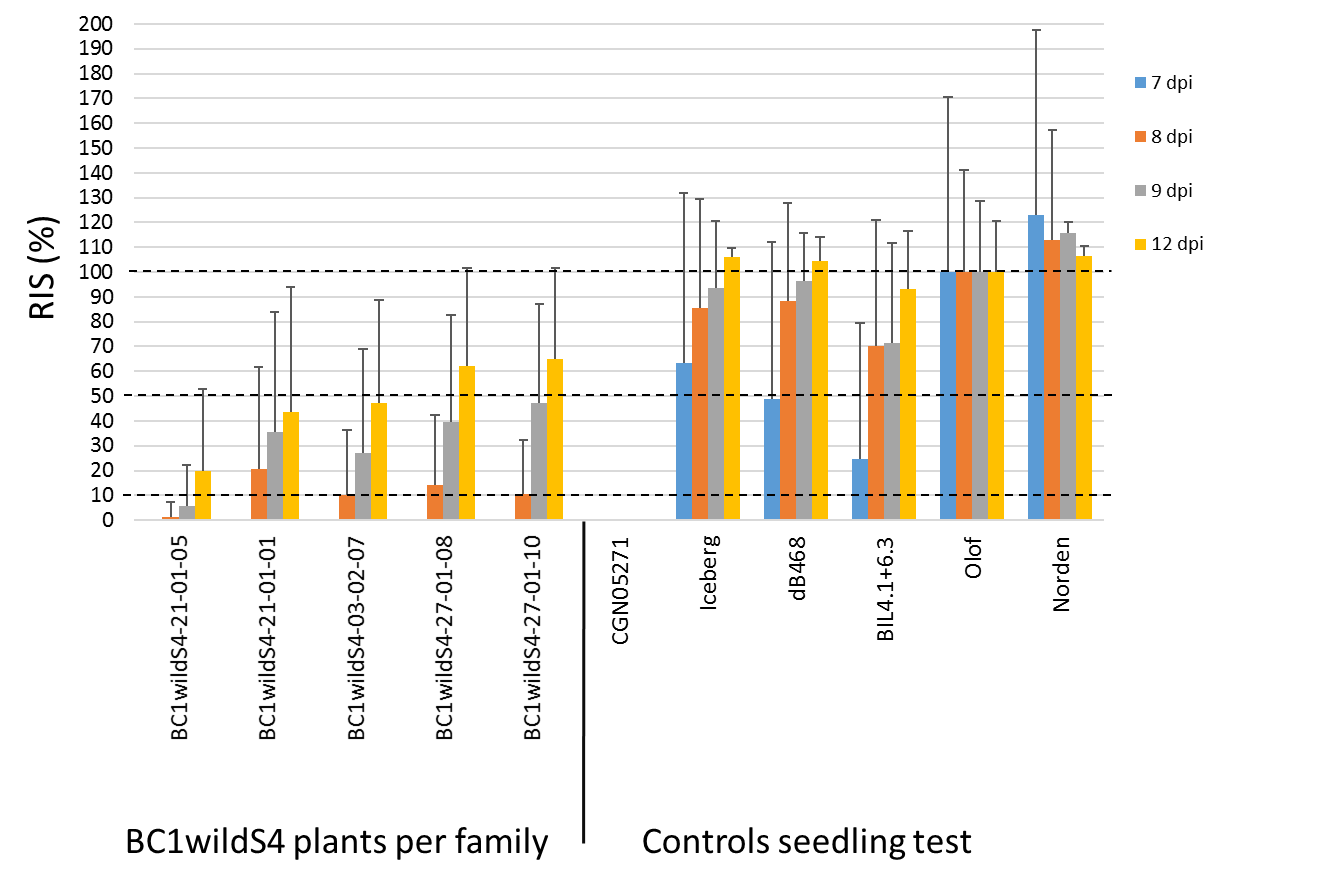


**Figure S13. Relative infection severity (RIS) at seedling stage in inbred offspring of five BC1wildS3 lineages selected for enhanced infection severity.** Remark: cv Iceberg and dB468 are control lines for reduced infection at adult plant stage, but not at seedling stage.

**
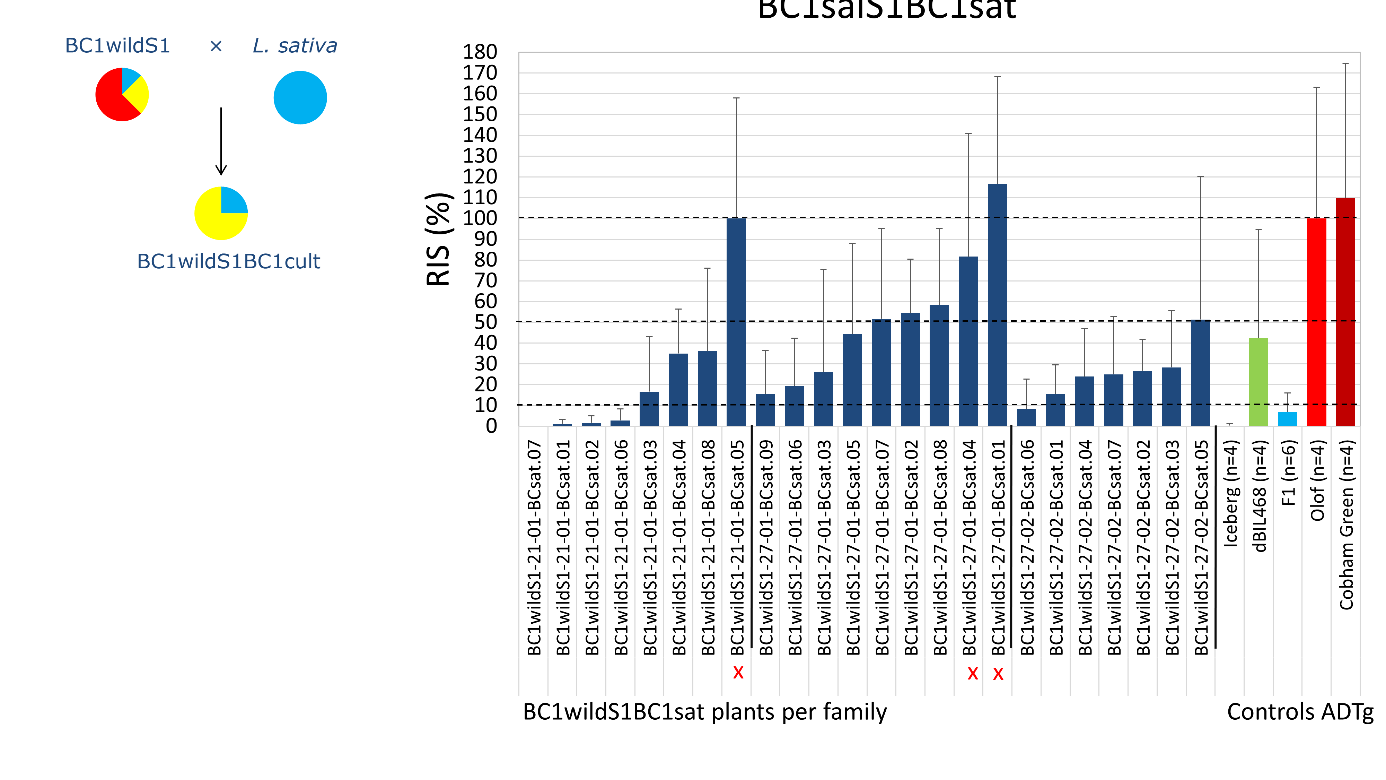
Figure S14. Crossing scheme to obtain BC1wildS1BC1cult plants from three BC1wildS1 founders and relative infection severity levels to Bl:21 of ‘BC1wildS1BC1cult’ families in an adult plant disease test (ADTg).** Pie charts depict the average genotypic composition of each generation. Red: homozygous *L. saligna*, blue: homozygous *L. sativa*, yellow: heterozygous. Different families are separated by vertical lines. Red crosses indicate plants selected for genotyping.
